# Multiplex Epstein-Barr virus *BALF2* genotyping detects high-risk variants in plasma for population screening of nasopharyngeal carcinoma

**DOI:** 10.1186/s12943-022-01625-6

**Published:** 2022-07-28

**Authors:** Jacob A. Miller, Malaya K. Sahoo, Fumiko Yamamoto, ChunHong Huang, Hannah Wang, James L. Zehnder, Quynh-Thu Le, Benjamin A. Pinsky

**Affiliations:** 1 Department of Radiation Oncology, Stanford University School of Medicine, 875 Blake Wilbur Drive, Palo Alto, CA 94304 USA; 2 Department of Pathology, Stanford University School of Medicine, 3375 Hillview Avenue, Palo Alto, CA 94304 USA; 3 Division of Infectious Diseases and Geographic Medicine, Department of Medicine, Stanford University School of Medicine, 300 Pasteur Drive, Stanford, CA 94305 USA

**Keywords:** Nasopharyngeal carcinoma, Epstein-Barr virus, Cancer screening, Plasma, BALF2, Cost-effectiveness

## Abstract

**Background:**

Epstein-Barr Virus (EBV)-associated nasopharyngeal carcinoma (NPC) exhibits unusual geographic restriction despite ubiquitous lifelong infection. Screening programs can detect most NPC cases at an early stage, but existing EBV diagnostics are limited by false positives and low positive predictive value (PPV), leading to excess screening endoscopies, MRIs, and repeated testing. Recent EBV genome-wide association studies (GWAS) suggest that EBV *BALF2* variants account for more than 80% of attributable NPC risk. We therefore hypothesized that high-risk *BALF2* variants could be readily detected in plasma for once-lifetime screening triage.

**Methods:**

We designed and validated a multiplex genotyping assay to detect EBV *BALF2* polymorphisms in human plasma. Targeted next-generation sequencing was used to validate this assay, conduct association studies with clinical phenotype, and longitudinally genotype plasma to assess within-host haplotype stability. We examined the association between NPC and *BALF2* haplotypes in a large non-endemic population and three prior EBV GWAS. Finally, we estimated NPC mortality reduction, resource utilization, and cost-effectiveness of *BALF2* variant-informed screening using a previously-validated cohort model.

**Results:**

Following analytical validation, the *BALF2* genotyping assay had 99.3% concordance with sequencing in a cohort of 24 NPC cases and 155 non-NPC controls. *BALF2* haplotype was highly associated with NPC in this non-endemic population (I613V: odds ratio [OR] 7.9; V317M: OR 178.8). No other candidate *BALF2* polymorphisms were significantly associated with NPC or hematologic disorders.

Longitudinal genotyping revealed 97.8% within-host haplotype concordance, indicative of lifelong latent infection. In a meta-analysis of 755 NPC cases and 981 non-NPC controls, *BALF2* I613V and V317M were significantly associated with NPC in both endemic and non-endemic populations.

Modeled variant-informed screening strategies achieved a 46% relative increase in PPV with 7% decrease in effective screening sensitivity, thereby averting nearly half of screening endoscopies/MRIs among endemic populations in east/southeast Asia.

**Conclusions:**

EBV *BALF2* haplotypes are temporally stable within hosts and can be readily detected in plasma via an inexpensive multiplex genotyping assay that offers near-perfect sequencing concordance. In endemic and non-endemic populations, I613V and V317M were highly associated with NPC and could be leveraged to develop variant-informed screening programs that mitigate false positives with small reductions in screening sensitivity.

**Supplementary Information:**

The online version contains supplementary material available at 10.1186/s12943-022-01625-6.

## Background

Epstein-Barr Virus (EBV)-associated nasopharyngeal carcinoma (NPC) is unusually restricted to certain regions and populations despite nearly ubiquitous EBV infection early in life [1]. NPC is the second-leading cause of head/neck cancer mortality worldwide, and has no definite modifiable risk factors [2]. Without biomarker-based screening, most patients present with NPC at an advanced stage and have worse prognoses despite treatment intensification [3].

Screening high-risk populations can detect most NPC cases at an early stage, but existing serologic and molecular diagnostics are limited by low positive predictive value (PPV) secondary to benign EBV reactivation [4, 5]. These false positives result in excess screening imaging, endoscopies, biopsies, and/or repeated laboratory testing which increase screening costs and visits. Ancillary triage testing with nasopharyngeal EBV PCR and plasma EBV next-generation sequencing (NGS) can increase PPV [6, 7]. However, each screening algorithm has certain advantages and disadvantages with respect to performance, referral rates, cost, and complexity.

Human genome-wide association studies (GWAS) have previously identified susceptibility loci which are associated with NPC risk [8]. However, the effect sizes are modest relative to the marked variation in NPC incidence worldwide. In contrast, several recent EBV GWAS have identified viral polymorphisms with much greater attributable risk [9–11]. In particular, two non-synonymous polymorphisms within the EBV *BALF2* gene (I613V, V317M) may contribute more than 80% of attributable risk in southern China. Because humans typically establish a single lifelong latent EBV infection, *BALF2* genotyping could serve as an adjunctive tool for once-lifetime screening triage [9]. We therefore hypothesized that a noninvasive molecular diagnostic could detect high-risk EBV *BALF2* variants in plasma and could serve to triage individuals for further screening work-up while remaining cost-effective in high-risk populations.

## Methods

### Multiplex BALF2 genotyping assay design

We designed a multiplex allele-specific real-time polymerase chain reaction (qPCR) genotyping assay to detect three non-synonymous polymorphisms in the EBV *BALF2* gene (NCBI RefSeq NC_007605.1 Aug 2018: V700L [162215C > A], I613V [162,476 T > C], V317M [163364C > T]). To permit single reaction multiplexing, we designed three conserved primer sets flanking the single nucleotide variants (SNVs), with one allele-specific propynyl-modified dual-labeled hydrolysis probe for each SNV (Biosearch Technologies, Petaluma, USA*)*. A fourth allele-specific probe detecting the wild-type V700 allele (162215C) served as an additional internal control for samples lacking these polymorphisms (Fig. S1, Table S1).

Recognizing the potential for off-target polymorphisms in primer/probe regions, on November 23, 2021 we identified 1050 EBV GenBank sequences aligning to the EBV *BALF2* region of interest (NC_007605.1:162115–163,464) with ≥98% coverage. Each primer was conserved in ≥98.7% of sequences. The 162215C, 162215C > A, 162476 T > C, and 163364C > T alleles were present in 78.3, 20.9, 37.2, and 29.2% of sequences, respectively.

Two synthetic dsDNA gene fragments (gBlocks, Integrated DNA Technologies, Coralville, USA) served as either the NPC risk-associated (V700, I613V, V317M) or non-risk-associated (V700L, I613, V317) controls (Table S2). Supernatant from the EBV-infected B95–8 cell line served as an additional wild-type whole-virus control (ATCC, Catalog #VR-1492). Further methodological details are available in the Supplementary Methods and Tables S1–3. Assay interpretation and example amplification curves are presented in Table S4 and Fig. S1.

### BALF2 genotyping qPCR analytical validation

The 95% lower limit of detection (LLOD) was assessed in replicates of 20 from 0.1–5.0 copies/μL template (1.0–50.0 copies/reaction) using the risk and non-risk dsDNA controls. Any amplification crossing the fluorescence threshold was regarded as detection. Linearity was assessed from 0.0 to 6.0 log10 copies/μL template in replicates of three. Because a minority of individuals may be latently infected with multiple distinct EBV variants, we evaluated the assay’s performance with mixed risk and non-risk dsDNA controls ranging from 0 to 100% allele frequency at a fixed total template concentration of 100 copies/μL in replicates of three.

### Clinical specimens

This study included human plasma specimens collected between July 1, 2019 and November 1, 2020 as part of routine clinical care for detection of EBV *EBNA-1* by qPCR. Clinical EBV DNA qPCR was conducted as previously described [12, 13]. Approximately 3 mL whole blood was collected in EDTA tubes, centrifuged, and at least 1.25 mL plasma aliquoted into separate tubes within six hours of collection. Total nucleic acids were extracted from 1000 μL plasma using the QIAsymphony DSP Virus/Pathogen Midi kit and eluted into 60 μL buffer AVE. After development and analytical validation of our genotyping qPCR, we retrospectively genotyped specimens meeting the following criteria: 1) EBV positive by EBNA-1 qPCR (C_t_ ≤ 45), 2) ≥20 μL residual extracted nucleic acid, and 3) highest viral load for a given patient within the study period. No diagnoses or indications for testing were excluded. Specimens were collected from patients with a range of benign and neoplastic EBV-associated disorders (Table S5).

### NGS validation of BALF2 genotyping qPCR

We validated the genotyping qPCR assay with targeted NGS using a subset of specimens from NPC cases and controls. We sequenced a region of the *BALF2* gene (NC_007605.1.162126–163,483) spanning the three non-synonymous polymorphisms of interest (Supplementary Methods). Sequences with a depth of at least 10 reads at the three SNV positions of interest were accepted for interpretation. We filtered out variants with the parameter ‘QUAL<30 | MQ<40 | DP<10 | MQ0F>4 | DV<3’. Specimens selected for sequencing were either the highest viral load specimen for a given patient or were specimens with residual extracted nucleic acid included in the longitudinal sequencing subset described below.

### Within-host longitudinal genotyping

To assess whether EBV *BALF2* haplotypes persisted over time, we longitudinally genotyped plasma specimens collected over the study period from a subset of individuals with multiple EBV-positive specimens.

### Modeled NPC mortality and resource utilization with variant-informed screening strategies

We estimated population-level NPC mortality reduction, resource utilization, and cost-effectiveness of *BALF2* variant-informed screening strategies using a previously-validated time-inhomogeneous decision-analytic cohort model (Table S6) [14]. This analysis was limited to high-risk populations with endemic NPC in southern China and southeast Asia. First, we conducted a meta-analysis of three prior EBV GWAS to model *BALF2* haplotype prevalence among NPC cases and non-NPC controls [9–11]. Thereafter, we compared variant-agnostic screening strategies from prospective studies to variant-informed screening strategies which triage positive plasma/nasopharyngeal EBV DNA with the *BALF2* genotyping qPCR. Full details regarding the model framework, population selection, screening strategies, and sensitivity analyses are detailed in the Supplementary Methods and Tables S6–11.

### Statistical analysis

Positive percent agreement (PPA) and negative percent agreement (NPA) were reported with Clopper-Pearson score 95% binomial confidence intervals using NGS as the reference method. The 95% LLOD was calculated using probit regression for each target. Linear regression was used to fit C_t_ values against nominal concentrations. Odds ratios for high-risk haplotypes (C-C-T and/or C-C-C at positions 162,215–162,476-163,364) were calculated using the common low-risk haplotypes as reference (sum of A-T-C and C-T-C). For EBV-positive NPC cases, the reference group includes all non-NPC controls for each individual study (present cohort, Xu et al., Hui et al., Lam et al.) [9–11]. Fisher exact tests were used to calculate *p-*values for SNV and haplotype associations with NPC. For targeted NGS, the *p*-value threshold for statistical significance was adjusted for the number of evaluated positions using the Bonferroni correction (α = 3.68 × 10^− 5^). Analyses were conducted using the *R* statistical software package.

## Results

### High-risk EBV variants are readily detected in plasma via a single-reaction genotyping assay

**Fig. 1:**
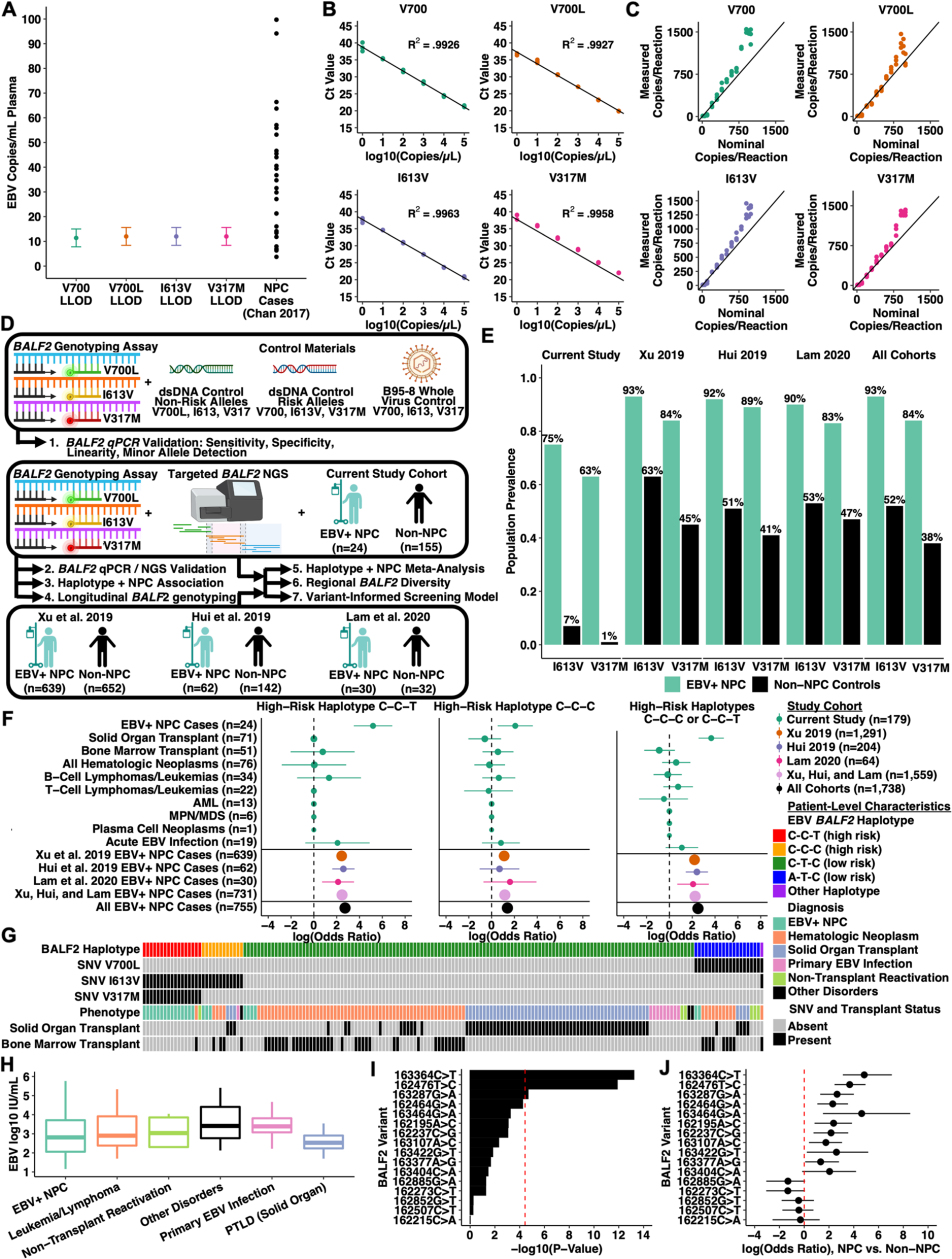
Multiplex EBV *BALF2* genotyping qPCR design, validation, and association studies with nasopharyngeal carcinoma in endemic and non-endemic populations. **A** Analytical sensitivity for each of the four *BALF2* qPCR targets. The 95% lower limit of detection with 95% confidence interval is reported for each target in units of EBV copies/mL plasma. In conjunction with the LLODs, the corresponding plasma viral load for 34 screen-detected preclinical NPC cases is presented to indicate likelihood of genotyping success. **B** Analytical linearity for each of the four *BALF2* qPCR targets, plotting cycle threshold (C_t_) against nominal dsDNA control concentration in units of log10 copies/μL template. **C** Mixing studies at fixed total template concentration (100 copies/μL template) combining high-risk and low-risk dsDNA controls, demonstrating detection of minor allele fractions as low as 10% for each of the four targets. Measured concentration is plotted against nominal concentration. In the presence of mixed alleles, the assay is approximately linear as allele fraction decreases. **D** Study overview and experimental workflow. First, the multiplex *BALF2* genotyping assay was analytically validated using synthetic dsDNA controls and wild-type B95–8 whole virus control. Next, our non-endemic cohort of 24 NPC cases and 155 non-NPC controls contributed to *BALF2* qPCR/NGS validation, longitudinal *BALF2* genotyping, and *BALF2-*NPC association. Finally, our non-endemic cohort and three predominantly endemic cohorts contributed to a meta-analysis of 755 EBV+ NPC cases and 981 non-NPC controls. This validated the association between *BALF2* haplotypes and NPC in multiple cohorts, further defined regional EBV genomic diversity, and was used to develop a variant-informed screening model. **E** Prevalence of I613V and V317M between EBV+ NPC cases and non-NPC controls in the present study and in the three prior EBV GWAS cohorts. **F** Log-transformed odds ratios with 95% confidence intervals for association between *BALF2* high-risk haplotypes (C-C-T, C-C-C, or both) and EBV+ NPC or other EBV-associated diseases in the current cohort and in the three prior EBV GWAS cohorts. **G** Individual patient characteristics from current study of 24 NPC cases and 155 non-NPC controls. *BALF2* haplotypes are defined by presence or absence of V700L, I613V, and/or V317M, which are associated with clinical phenotype. **H** Plasma EBV viral load (log10 IU/mL plasma) across phenotypes of 155 patients included in current study, demonstrating no significant difference between plasma viral load and phenotype. **I** and **J** Association between NPC and other BALF2 single nucleotide variants identified by next-generation sequencing. Log-transformed *P-*value from association test and log-transformed odds ratios with 95% confidence intervals are presented for three variants of interest (V700L = 162215C > A, I613V = 162476C > T, V317M = 163364C > T) and 13 additional variants differentially associated with NPC. V700L is mutually exclusive with I613V and V317M, was rare in this population, and was not associated with NPC risk. Only one other variant (163287G > A, synonymous) exceeded the Bonferroni-corrected *P-*value threshold

We designed and validated a multiplex allele-specific real-time polymerase chain reaction (qPCR) genotyping assay to detect three EBV *BALF2* variants (V700L, I613V, V317M; Supplementary Methods, Fig. S1, Tables S1–4). The wild-type V700 allele was selected as an internal control for samples lacking these polymorphisms. The assay’s 95% lower limit of detection was 2.0 copies/reaction (95% CI 1.4–2.6) with < 20% coefficient of variation across six orders of magnitude (R^2^ ≥ 0.992, Fig. 1A-B, Tables S12–13). Non-specific amplification was not observed for off-target alleles, and replicates of the B95–8 wild-type whole-virus control also confirmed specificity. In mixing experiments ranging from 0 to 100% allele frequency, the assay detected allele frequencies as low as 10% for each of the four targets, below the host heterozygosity threshold (Fig. 1C, Table S14) [9].

### Multiplex BALF2 genotyping qPCR has near-perfect concordance with next-generation sequencing

We sequenced the *BALF2* region in 258 clinical plasma specimens genotyped by qPCR, and 152 had adequate sequencing depth and coverage (Supplementary Methods). Samples with adequate sequencing depth and coverage had higher viral load (median 1600 vs. 201 IU/mL, *p* < 0.01). There was a single discordant genotyping call between qPCR and NGS. In a 43-year-old immunosuppressed woman with heart/lung transplantation, the sixth of six plasma specimens collected over 4.9 months showed qPCR loss of I613V which was detected on all five prior specimens. The specimen was sequenced and revealed the I613V mutation in 35/36 (97.2%) reads, reflecting false negative qPCR, possibly due to low viral load (EBNA-1 < 100 IU/mL). Positive and negative percent agreements for V700L, I613V, and V317M were otherwise 100%, and overall haplotype concordance between qPCR and NGS was 99.3% (151/152, Table S15).

### BALF2 haplotypes are associated with NPC in a non-endemic cohort

We genotyped plasma specimens from 179 unique patients in a non-endemic population, including 155 non-NPC controls and 24 EBV-positive NPC cases (Table S5, Fig. 1G). Among controls, the most common indication for plasma EBV PCR was monitoring after solid organ transplant (44%) or bone marrow transplant (33%). Seventy-six control patients (49%) had hematologic neoplasms with (66%) or without (33%) prior bone marrow transplant, including EBV-positive lymphomas/leukemias. Nineteen patients (12%) had no history of transplant or neoplasm, including ten patients with primary EBV infection. There was no significant association between plasma EBV EBNA-1 viral load and disease phenotype (Fig. 1H).

High-risk *BALF2* haplotypes, defined by the presence of I613V with or without V317M, were rare among non-NPC controls (Fig. 1D-E, Table S16). The C-C-C and C-C-T high-risk haplotypes were present in 5.8 and 1.3% of controls, compared with 12.5 and 62.5% of NPC cases. Using the low-risk A-T-C and C-T-C haplotypes as reference, both the C-C-C (odds ratio [OR] 7.9 95% confidence interval [CI] 1.7–37.1) and C-C-T (OR 178.8, 95% CI 33.1–965.3) haplotypes were highly associated with NPC in this non-endemic population (Fig. 1F, Table S16). We observed no association between these haplotypes and other diseases, including hematologic neoplasms.

### BALF2 haplotypes are associated with NPC in a meta-analysis of endemic and non-endemic cohorts

In a meta-analysis of 755 NPC cases and 981 non-NPC controls from this study and three previously-published EBV GWAS, the NPC odds ratios for the C-C-C and C-C-T haplotypes were 4.0 (95% CI 2.6–6.0) and 15.4 (95% CI 11.2–21.0), respectively (Fig. 1D-F, Table S16). While I613V and V317M were common (> 75%) in NPC cases across cohorts, they were uncommon in non-endemic controls (7.1%) relative to endemic controls (60.5%), suggesting that variable NPC incidence could be explained by underlying *BALF2* haplotype prevalence.

We also evaluated the association between clinical phenotypes and other *BALF2* SNVs. For example, the previously-described 162507C > T and 162852G > T synonymous polymorphisms have been rarely observed (3%) in NPC cases but are common in endemic controls (41–43%). Among 108 unique patients with sequenced specimens, neither mutation was significantly associated with NPC. Beyond I613V and V317M, only the synonymous 163287G > A SNV reached statistical significance (Fig. 1I-J, Table S17). We observed no *BALF2* SNVs which were significantly associated with EBV-positive leukemias/lymphomas or post-transplant lymphoproliferative disorders. We also assessed whether other SNVs were associated with high-risk *BALF2* haplotypes, and identified multiple variants which were significantly correlated with I613V and V317M. For example, seven BALF2 SNVs occur with 100% frequency in the I613V/V317M haplotype and with 0–2% frequency in low-risk haplotypes (*p* ≤ 1.31 × 10^− 7^). This supports the hypothesis that high-risk EBV variants are transmitted locally rather than developing de novo after primary infection (Table S18).

### Longitudinal genotyping within hosts confirms temporal stability of BALF2 haplotypes

**Fig. 2:**
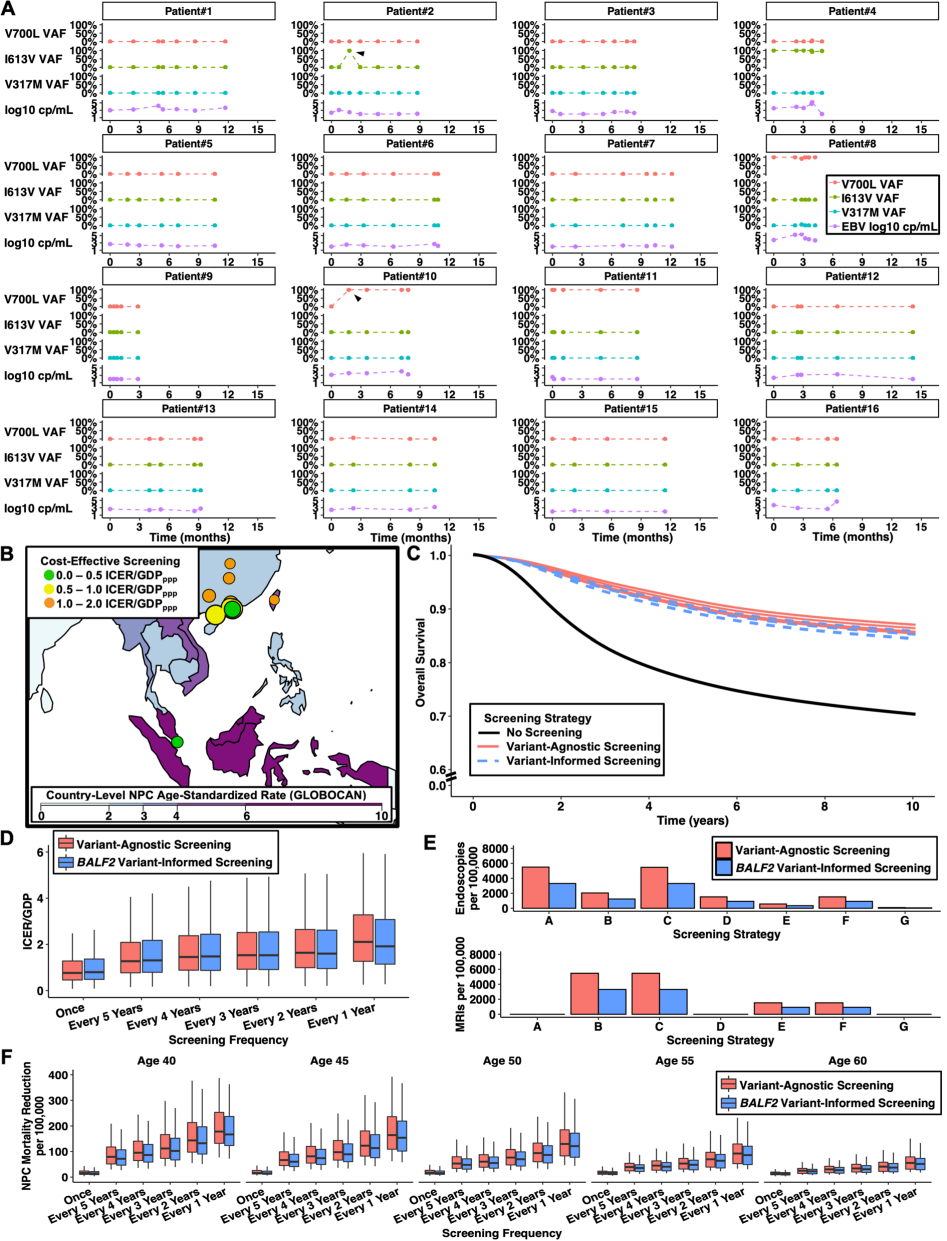
Longitudinal EBV *BALF2* genotyping and modeled variant-informed NPC screening strategies in 12 high-risk endemic populations. **A** A subset of 16 patients with serial EBV-positive plasma specimens were subject to *BALF2* genotyping by qPCR and NGS to assess temporal haplotype stability. Variant allele fraction (VAF) is plotted against time from first specimen collection for the three qPCR targets (V700L, I613V, V317M). The sample’s viral load in log10 EBNA-1 IU/mL is plotted below the allele frequencies. Two patients had one specimen each with temporally-discordant haplotypes. Patient #2 was a lung/liver transplant recipient with I613V detected in only the third of seven plasma specimens collected over 8.7 months. Patient #10 was a kidney transplant recipient with large-cell lymphoma who had V700 detected only in the first of five specimens collected over 7.8 months, whereas the subsequent four specimens harbored V700L, possibly indicating mutagenesis. **B** Map of east/southeast Asia with 12 included high-risk populations. Shading represents the national NPC incidence rate. Each bubble indicates a single population with size proportional to incidence rate. Bubble color indicates the cost-effectiveness of variant-informed screening at variable willingness-to-pay thresholds. **C** Modeled survival in a hypothetical cohort of 50-year-old patients in southern China. Survival differs with no screening (black line), seven variant-agnostic screening strategies (red solid lines), and seven variant-informed screening strategies (blue dashed lines) due to weighted stage distributions dictated by effective screening sensitivity. **D** Cost-effectiveness of variant-agnostic and variant-informed screening strategies across variable screening frequencies. Box plots indicate median with interquartile range. **E** Resource utilization after initial biomarker screening for variant-agnostic (A_0_-G_0_) and variant-informed screening strategies (A_BALF2_-G_BALF2_). Bar charts indicate absolute number of screening endoscopies and MRIs per 100,000 screened subjects. Referrals for endoscopy/MRI decrease after triage with *BALF2* qPCR. **F** NPC deaths per 100,000 screened individuals with variable screening frequencies and initial screening ages

Because EBV establishes lifelong latent infection, *BALF2* genotyping could facilitate once-lifetime screening triage. To assess whether EBV *BALF2* haplotypes persisted over time, we genotyped 90 EBV-positive plasma specimens collected from a subset of 16 patients. These patients had a median of 5 (range, 2–7) specimens genotyped over a median period of 8.6 months (range, 2.8–13.9). Among the 90 genotyped specimens, 88 (97.8%) haplotype calls were concordant within a given individual over time (Fig. 2A). The two discordant specimens both occurred in individuals with solid organ transplantation (Fig. 2A, Patients #2 and #10) and may represent mutagenesis under immunosuppression or reactivation of distinct latent infections from the host and donor tissue.

### Variant-informed NPC screening strategies reduce false positives and unnecessary procedures

We estimated population-level NPC mortality reduction, resource utilization, and cost-effectiveness of *BALF2* variant-informed screening strategies using a previously-validated time-inhomogeneous decision-analytic cohort model (Fig. S2) [14]. Full details regarding the model framework, population selection, screening strategies, and sensitivity analyses are provided in the Supplementary Methods and Tables S6–9.

First, we conducted a meta-analysis of three prior EBV GWAS to model endemic *BALF2* haplotype prevalence among NPC cases and non-NPC controls [9–11]. Thereafter, we compared seven variant-agnostic screening strategies from prospective studies to seven variant-informed strategies wherein positive plasma/nasopharyngeal EBV PCR are triaged using the *BALF2* genotyping qPCR (Table S10). Twelve high-risk populations in southern China, Hong Kong SAR, Macao SAR, Republic of China, and Singapore met inclusion criteria (Fig. 2B, Table S11).

Variant-informed screening increased PPV by a median of 46% (range, 26–51%) with an absolute decrease in screening sensitivity of 7%. Variant-informed screening reduced referrals for endoscopy and/or MRI by approximately 40% relative to the corresponding variant-agnostic strategy (Table S10). This reduction in referrals for further screening steps averted a median of 2969 screening visits per 100,000 subjects (Table S19).

For a hypothetical cohort of 50-year-old men and women who develop NPC in southern China, 10-year survival improved from 70.4% (95% CI 68.1–72.5%) in an unscreened cohort to a median of 85.7% (range, 85.4–87.0%) with variant-agnostic screening and 85.2% (range, 84.3–85.9%) with variant-informed screening (Fig. 2C, Table S19). In the highest incidence region, the small reduction in screening sensitivity after *BALF2* triage resulted in approximately 3.4 excess NPC deaths and 600 fewer false-positives requiring endoscopy/MRI per 100,000 subjects screened.

### Variant-informed NPC screening is cost-effective and facilitates once-lifetime testing

The base case screened adult men and women once at age 50 years (Fig. 2D, Table S19–20). Variant-informed screening was cost-effective in all populations except Hengdong, China (due to lower NPC incidence). Across the 12 populations and 14 screening strategies, an initial screening age of 40–45 tended to be most cost-effective irrespective of screening interval (Table S21). Screening intervals as short as every two years could be cost-effective. Variant-informed screening became more cost-effective as the number of lifetime screens increased due to the increasing proportion of subjects known to have low-risk *BALF2* haplotypes that were never subsequently screened (Fig. S3). Sensitivity analysis identified parameters that most impacted cost-effectiveness (Fig. S3, Tables S22–23).

## Discussion

Existing NPC screening strategies typically utilize EBV serology or plasma PCR as the initial screening assay [4–7, 10]. These programs achieve PPV ranging from 2 to 16% and do not currently leverage EBV genotyping to mitigate false positives. In light of existing laboratory screening infrastructure, we developed and validated an inexpensive single-reaction molecular diagnostic to detect high-risk EBV *BALF2* haplotypes. High-risk variants were readily detectable in human plasma and were longitudinally stable within hosts. This assay had excellent analytical performance, near-perfect NGS concordance, and typically offered higher sensitivity than NGS. While prior EBV GWAS have genotyped tumor tissue or oropharyngeal swabs, our study demonstrates the feasibility of genotyping plasma EBV DNA. Modeled variant-informed screening strategies remained highly cost-effective with a 7% absolute decrease in screening sensitivity (< 1% decrement in 10-year survival) and an approximate 40% decrease in referrals.

At least three recent endemic EBV-NPC GWAS have been conducted. Notably, each study observed an association between NPC and the two high-risk *BALF2* haplotypes, with similar effect sizes (odds ratio 7.9–11.1) and proportion of NPC cases (90–93%). The largest study was conducted by Xu et al. and served as the basis for selection of I613V and V317M as qPCR targets [9]. In Southern China, these variants account for 83% of the overall NPC risk, far exceeding loci in the human genome [8]. Susceptibility loci in EBV *EBER2* have also been recently identified in the Hong Kong population, but these did not meet statistical significance in Xu et al. and were not evaluated in our study [10, 11]. In contrast, a recent study of 47 Japanese patients with EBV-positive NPC reported that none harbored the I613V + V317M haplotype, whereas 21% were positive for I613V alone [15]. Seven SNVs were specifically associated with non-endemic Japanese NPC. While I613V and V317M are common (60%) in endemic non-NPC controls, they are rare (12.6%) in non-endemic East Asia, Africa, and Western countries, which was replicated in our non-endemic population (7.7%) and in the Japanese cohort (9.6%). Collectively, these studies indicate that high-risk BALF2 variants are common in endemic regions but are not necessarily required for NPC carcinogenesis, and that regional EBV genomic diversity may explain differential NPC risk. Thus far, in vitro studies have implicated BALF2 variants in facilitating lytic reactivation; further functional studies are warranted to better understand the impact of I613V/V317M on NPC carcinogenesis [9, 15].

Modeling approaches can aid local healthcare policymakers and epidemiologists to determine the optimal balance between screening resources, complexity, and performance. If *BALF2* genotyping were incorporated into screening algorithms, laboratories could consider screening with a single multiplex qPCR detecting *BamHI-W, BALF2* V317M, *BALF2* I613V, and a single-copy conserved target. This approach would require minimal additional laboratory costs and could decrease referrals for subsequent screening steps by approximately 40%. While a multiplex genotyping qPCR is advantageous in its low cost/complexity, we anticipate superior discrimination with more complex NGS-based variant panels [10]. Although not evaluated in our study, once-lifetime EBV genotyping using oropharyngeal specimens warrants further evaluation.

There are multiple limitations to our study. First, we validated our assay using specimens from a non-endemic population that had few healthy controls. Although we observed no association between *BALF2* haplotypes and other non-NPC diseases, it is possible that non-NPC controls had different haplotype prevalence relative to the healthy population. Second, the ability to triage individuals once in their lifetime with *BALF2* genotyping is predicated on haplotype stability over time and absence of multiple EBV co-infections. Because longitudinal specimens were not available over a years- or decades-long period, it is uncertain whether haplotypes may change over longer time scales. Third, *BALF2* haplotype distributions for NPC cases and controls were derived from a meta-analysis of three studies which predominantly included subjects in southern China. The degree to which these distributions vary within southeast Asia is unknown, and would impact effective screening sensitivity.

## Conclusions

Approximately 93% of endemic nasopharyngeal carcinoma harbors high-risk EBV *BALF2* haplotypes. These haplotypes are stable over time within hosts and readily detectable in plasma using an inexpensive single-reaction multiplex genotyping assay. The *BALF2* I613V and V317M polymorphisms are rare in non-endemic controls, supporting the hypothesis that regional EBV genomic diversity contributes to differential NPC risk worldwide. Triaging subjects who test positive for plasma/nasopharyngeal EBV DNA using *BALF2* genotyping could substantially reduce referrals for more complex and expensive endoscopy/MRI. Across seven prospectively-evaluated screening strategies in 12 high-risk endemic populations, these variant-informed strategies maintain high screening sensitivity while averting 40% of referrals for endoscopy/MRI. In suitable populations, this may be a low-cost and readily accessible alternative to higher-complexity triage algorithms, and could identify low-risk individuals who require no further lifetime screening.

## Supplementary Information

**Additional file 1.**

## Data Availability

All data generated or analyzed during this study are included in this published article and its additional files. Sequence data that support the findings of this study have been deposited in the NCBI Sequence Read Archive with the primary accession code PRJNA848410.

## Abbreviations

AML: acute myeloid leukemia
C_t_: cycle threshold
dsDNA: double-stranded DNA
EBV: Epstein-Barr Virus
ELISA: enzyme-linked immunosorbent assay
GDP_ppp_: purchasing power parity-adjusted per-capita gross domestic product
GWAS: genome-wide association study
ICER: incremental cost-effectiveness ratio
IMRT: intensity-modulated radiotherapy
IU: international units
LLOD: lower limit of detection
MPN/MDS: myeloproliferative neoplasm / myelodysplastic syndrome
MRI: magnetic resonance imaging
NED: no evidence of disease
NGS: next-generation sequencing
NPA: negative percent agreement
NPC: nasopharyngeal carcinoma
OS: overall survival
PCR: polymerase chain reaction
PPA: positive percent agreement
PPV: positive predictive value
qPCR: real-time polymerase chain reaction
SNV: single nucleotide variant
VAF: variant allele fraction
WHO: World Health Organization
WTP: willingness-to-pay
