# Supplementary material for: Multiplex Epstein-Barr virus BALF2 genotyping detects high-risk variants in plasma for population screening of nasopharyngeal carcinoma
Source: Mol Cancer. 2022 Jul 28;21:154. doi: 10.1186/s12943-022-01625-6 (PMC9330640; doi:10.1186/s12943-022-01625-6)

Supplementary Methods

Multiplex BALF2 Genotyping Assay Design
We designed a multiplex allele-specific real-time polymerase chain reaction (qPCR) genotyping assay to detect three non-synonymous polymorphisms in the EBV *BALF2* gene (NCBI RefSeq NC_007605.1 Aug 2018: V700L [162215C>A], I613V [162476T>C], V317M [163364C>T]). We selected the wild-type V700 allele (162215C) to serve as an additional internal control for samples lacking any of these polymorphisms.

To permit single reaction multiplexing, we designed three conserved primer sets flanking the single nucleotide variants (SNVs), with one allele-specific propynyl-modified dual-labeled hydrolysis probe for each SNV (Biosearch Technologies, Petaluma, USA). A fourth allele-specific probe detecting V700 served as an internal control (Figure S1). Each of the four allele-specific probes was designed to maximize the mismatch ΔTm while maintaining probe specificity and a sufficiently high annealing temperature (Supplementary Table S1). Recognizing the potential for off-target polymorphisms in primer/probe regions, on November 23, 2021 we identified 1,050 EBV GenBank sequences aligning to the EBV *BALF2* region of interest (NC_007605.1:162115-163464) with ≥98% coverage. Each primer was conserved in ≥98.7% of sequences. The 162215C, 162215C>A, 162476T>C, and 163364C>T alleles were present in 78.3%, 20.9%, 37.2%, and 29.2% of sequences, respectively.

Two synthetic dsDNA gene fragments (gBlocks, Integrated DNA Technologies, Coralville, USA) served as either the NPC risk-associated (V700, I613V, V317M) or non-risk-associated (V700L, I613, V317) controls (Supplementary Table S2). These controls were diluted in Tris-EDTA buffer (10 mM Tris, 1 mM EDTA). Supernatant from the EBV-infected B95-8 cell line served as an additional wild-type whole-virus control (ATCC, Catalog #VR-1492).

Real-time PCR was performed using 12.5 μL FastStart TaqMan Probe Master Mix (Roche, Switzerland), 2.0 μL primer/probe mix, 0.5 μL nuclease-free water, and 10.0 μL template (Supplementary Figure S1, Table S3). All experiments were conducted using a BioRad CFX96 real-time PCR instrument (BioRad, Hercules, CA, USA). Cycling conditions were 95°C for 4:00 and then 45 cycles of 95°C for 00:30, 63.0°C for 00:30, and 72°C for 00:30. All qPCR experiments included a no-template control (nuclease-free water), a wild-type whole-virus control (3.98×10^4 IU/mL B95-8 supernatant extracted into AVE buffer), synthetic dsDNA risk control (10^4 copies/μL template), and synthetic dsDNA non-risk control (10^4 copies/μL template). Annealing temperature was optimized with a temperature gradient. Fluorescence was collected in all channels. Fixed fluorescence thresholds of 200 relative fluorescence units ([RFU], V700-FAM), 100 RFU (V700L-CAL560), 300 RFU (I613V-CAL610), and 300 RFU (V317M-Q670) were used to determine each target's threshold cycle (C_t). Assay interpretation and example amplification curves are presented in Supplementary Table S4 and Figure S1.

BALF2 Genotyping qPCR Analytical Validation
The 95% lower limit of detection (LLOD) was assessed in replicates of 20 from 0.1-5.0 copies/μL template (1.0-50.0 copies/reaction) using the risk and non-risk dsDNA controls (Supplementary Table S12). Any amplification crossing the fluorescence threshold was regarded as detection. Linearity was assessed from 0.0 to 6.0 log10 copies/μL template in replicates of three (Supplementary Table S13). Because a minority of individuals may be latently infected with multiple distinct EBV variants, we evaluated the assay's performance with mixed risk and non-risk dsDNA controls ranging from 0-100% allele frequency at a fixed total template concentration of 100 copies/μL in replicates of three (Supplementary Table S14).

Clinical Specimens
This study included human plasma specimens collected between July 1, 2019 and November 1, 2020 as part of routine clinical care for detection of EBV *EBNA-1* by qPCR. Clinical EBV DNA qPCR was conducted as previously described.[1,2] Approximately 3 mL whole blood was collected in EDTA tubes, centrifuged, and at least 1.25 mL plasma aliquoted into separate tubes within six hours of collection. Total nucleic acids were extracted from 1000 μL plasma using the QIAsymphony DSP Virus/Pathogen Midi kit and eluted into 60 μL buffer AVE. Nucleic acid extraction and clinical testing were conducted at the Stanford Clinical Virology Laboratory, which serves tertiary-care academic hospitals and affiliated outpatient facilities in the San Francisco Bay Area.

After development and analytical validation of our genotyping qPCR, we retrospectively genotyped specimens meeting the following criteria: 1) EBV positive by EBNA-1 qPCR ($C_t \leq 45$), 2) ≥20 μL residual extracted nucleic acid, and 3) highest viral load for a given patient within the study period. No diagnoses or indications for testing were excluded. Specimens were collected from patients with a range of benign and neoplastic EBV-associated disorders (Supplementary Table S5). This study was conducted with Stanford University institutional review board approval.

NGS Validation of BALF2 Genotyping qPCR

We validated the genotyping qPCR assay with targeted NGS using a subset of specimens from NPC cases and controls (Supplementary Table S15). We sequenced a portion of the *BALF2* gene (NC_007605.1.162126-163483) spanning the three non-synonymous polymorphisms of interest. Specimens selected for sequencing were either the highest viral load specimen for a given patient or were specimens with residual extracted nucleic acid included in the longitudinal sequencing subset described below.

We designed 28 conserved primers to generate 14 small overlapping amplicons for targeted enrichment. Given the small insert size (median 153 bases, range 93-219), fragmentation was not performed and adapter sequences were included at the 5' end of each primer. Each of the 28 primers was separately synthesized with read 1 and read 2 adapters to facilitate paired-end sequencing. Even- and odd-numbered primersets were pooled separately.

Extracted nucleic acid was amplified by PCR in two reactions using 12.5 µL LongAmp Taq 2X Mastermix Hot Start (New England BioLabs, Ipswich, MA), 0.3125 µL of 2 µM read 1 odd or even-numbered primersets (25 nM each primer), 0.3125 µL of 2 µM read 2 odd or even-numbered primersets (25 nM each primer), 1.875 µL nuclease-free water, and 10.0 µL template. Target enrichment PCR conditions were 94°C for 0:30, and then 45 cycles of 94°C for 00:30, 57.0°C for 00:30, and 65°C for 01:00 prior to final 65°C extension for 10:00.

Libraries were prepared using NEBNext library preparation reagents for Illumina sequencing instruments (New England BioLabs, Ipswich, MA). Half of the products from the two PCR reactions (25 µL total) were pooled and purified with 45 µL (1.8X ratio) AMPure XP beads (Beckman Coulter, Brea, CA) and 80% ethanol, then resuspended into 25 µL AVE elution buffer. Libraries were then indexed using dual index primers in a 50 µL reaction containing 25 µL NEBNext® UltraTM II Q5® Master Mix (M0544), 1 µL index 1 (i7) primer, 1 µL index 2 (i5) primer, and 23 µL amplified DNA. Thermocycler settings for indexing were: 98°C for 00:30, then 12 cycles of 98°C for 00:10 and 65.0°C for 01:15 prior to final extension at 65.0°C for 05:00.

Indexed libraries were then purified with 90 µL (1.8X ratio) AMPure XP beads and 80% ethanol, then resuspended into 40 µL AVE elution buffer. Individual indexed specimens were then pooled and normalized (1-20uL each) based on EBNA-1 qPCR viral load. The pooled library was then purified with a 1:1 ratio of AMPure XP beads and 80% ethanol, then resuspended into 40 µL AVE elution buffer. Pooled indexed library fragment size was measured with a BioAnalyzer 2100. The library was then quantified using the Qubit dsDNA broad range assay and diluted to 15 pM. Each sequencing run contained one no template control, one wild-type B95-8 control, and up to 94 clinical specimens. Libraries were sequenced on an Illumina MiSeq using paired-end 150-cycle sequencing using the MiSeq Nano reagent kit V2.

Trimmed reads were assembled using the Burrows-Wheeler Aligner and variants were called using BCFTools using NC_007605.1 as reference. Sequences with a depth of at least 10 reads at the three SNV positions of interest were accepted for interpretation. We filtered out variants with the parameter 'QUAL<30 | MQ<40 | DP<10 | MQ0F>4 | DV<3'.

Modeled NPC Mortality and Resource Utilization with Variant-Informed Screening Strategies
We estimated population-level nasopharyngeal carcinoma (NPC) mortality reduction, resource utilization, and cost-effectiveness of *BALF2* variant-informed screening strategies using a previously-validated time-inhomogeneous decision-analytic cohort model (Supplementary Table S6).[3] This was limited to high-risk populations with endemic NPC in southern China and southeast Asia.

First, we conducted a meta-analysis of three prior EBV genome-wide association studies (GWAS) to model *BALF2* haplotype prevalence among NPC cases and non-NPC controls (Supplementary Table S16).[4–6] Thereafter, we compared variant-agnostic screening strategies from prospective studies to variant-informed screening strategies which triage positive plasma/nasopharyngeal EBV DNA with the *BALF2* genotyping qPCR (Supplementary Table S10).

<u>Study Populations and Incidence Data</u>
We selected 12 populations to compare variant-informed and variant-agnostic screening (Supplementary Table S23). These populations satisfied the following criteria:

1) Populations with endemic EBV-associated NPC.
2) Populations with known EBV *BALF2* haplotype distributions from association studies.
3) Populations in regions with ongoing or previously-conducted NPC screening programs.
4) Populations with available age and sex-specific incidence rates published in national cancer registries or in the World Health Organization's *Cancer Incidence in Five Continents* (*CI5 X-XI*).[7–12]
5) Populations with available megavoltage radiotherapy services as reported in the IAEA DIRAC database.[13]

Each population had economic characteristics and sufficiently high NPC incidence such that NPC screening was likely to be cost-effective as estimated in a prior study.[3] Populations in southern China (Hong Kong SAR, Macao SAR,

Guangdong, Guangxi, Hunan), Singapore, and the Republic of China met these criteria. The most recent incidence rates for these 12 populations were obtained from *CI5 X-XI,* the Hong Kong Cancer Registry, the Singapore Cancer Registry, and the Republic of China Cancer Registry[7,8,10–12,14], which were adjusted for the proportion of incident NPC cases in each region which were WHO type II/III (Supplementary Table S11). In Singapore, ethnicities with high NPC incidence were studied separately from pooled national data. For men and women, the age-standardized rates for these 12 populations ranged from 3.6-16.6 cases/100,000 life-years.

Decision Analytic Model

We utilized a previously-validated time-inhomogeneous decision-analytic cohort model to study NPC mortality reduction and resource utilization of *BALF2* variant-informed screening strategies.[3] Full details regarding development and validation of this model are available from the original publication and reports from prospective screening trials. Briefly, subjects enter model at the age of a single screening intervention and are followed until death from NPC or other causes over a lifetime horizon. Heath states include perfect health, five separate stages of undetected NPC (AJCC 7 stages I, II, III, IVA/B, IVC), five separate stages of detected incident NPC (AJCC 7 stages I, II, III, IVA/B, IVC), remission with no evidence of disease after definitive (chemo)radiotherapy, locoregional recurrence or distant metastasis under treatment with indefinite palliative chemotherapy, and death (Supplementary Figure S2).

Each AJCC 7 nonmetastatic stage of NPC (I-IVB) undergoes definitive therapy with radiotherapy or chemoradiotherapy, while *de novo* metastatic disease (IVC) undergoes treatment with indefinite palliative gemcitabine/platinum chemotherapy with or without one-time palliative radiotherapy. Transition probabilities between states were calibrated using epidemiologic estimates from incidence databases and from prospective screening studies identified in a systematic literature review.[3]

Patients diagnosed with nonmetastatic NPC undergo treatment and enter the remission state, and then could either die from background mortality or develop a locoregional and/or distant recurrence prior to death from NPC. Using the R *heemod* package, subjects who developed NPC enter the state of undiagnosed stage I disease and could progress to more advanced stages of undetected disease.[15] Alternatively, each undiagnosed stage of NPC could instead present symptomatically with detected NPC, which is identical to observed stage-specific incidence rates in unscreened populations. At the time of onetime screening, a true positive (based on strategy-specific sensitivity) results in immediate detection at that stage. False negatives and unscreened cases resulted in continued stage progression until usual symptomatic detection. For each screening step, a positive result (whether true positive or false positive) results in testing at the next screening step (e.g., nasoendoscopy after positive serology). There was also the usual risk of death or remaining in the same state for each given state.

For each population, background mortality was derived from WHO global health observatory country-, age- and sex-specific mortality rates.[16]

For the base case, men and women are screened once at age 50 years, which was closest to the median age in most screening studies. Interval screening, variable initial screening ages, and the exclusive screening of men were studied in sensitivity analyses. For interval screening, all screening ended after age 70.

Recurrence and Survival Estimates

Among patients with detected NPC (whether by screening or symptomatic presentation), stage-specific time-dependent transition probabilities to LRR, DM, or death were derived from survival models trained using extracted individual patient data from 9,864 individuals included in 17 studies and treated in China with standard-of-care intensity-modulated (chemo)radiotherapy (stages IVA/B) or gemcitabine/platinum chemotherapy (stage IVC) after MRI staging.[3] These stage-specific recurrence and survival models were then externally validated using individual patient data (n=729) extracted from nine studies from patients treated with IMRT and MRI staging outside of China. These 26 total studies were identified in a previously-conducted systematic review.[3]

Health State Utilities

Time-dependent health utilities during and after radiotherapy alone were derived from the experimental (IMRT) arm of a randomized trial of patients with NPC undergoing definitive radiotherapy alone (Supplementary Table S6).[17,18] Disutilities for the addition of chemotherapy to radiotherapy were derived from a series of patients with NPC treated in Taiwan who completed the EORTC QLQ-C30 questionnaires.[19] Health utilities for patients undergoing indefinite chemotherapy for locoregional or distant recurrence were derived from the control arm of the Checkmate 141 (week 15).[20] Responses from the EORTC QLQ-C30 were mapped to EQ-5D values using a model derived from a population of patients with head and neck cancers.[21]

For the base case, we assumed that subjects who screen as false positives by any strategy had a health state of 1.000 based on literature from lung cancer screening.[22] However, we also modeled the impact of a one-month decrement in

healthy utility to 0.900 for false positives based on quality of life from the breast cancer screening literature.[23] For true positives (detected NPC cases), we assigned a one-month pre-treatment decrement in health utility to 0.886 based on prior literature.[24] For the probabilistic sensitivity analysis, we created joint uncertainty distributions for health state utilities to respect preference order for certain health states over others based on base case values and clinical experience, as previously described.[3]

Workup, Staging, and Treatment Assumptions

Initial workup and management were based upon the National Comprehensive Cancer Network 2021 head and neck cancer guidelines (Supplementary Tables S7-9).[25] We assumed that patients who developed NPC underwent clinical evaluation by a physician, endoscopic examination, needle biopsy, pathology review, CT and MRI of the head and neck, and CT of the chest to evaluate for metastatic disease (Supplementary Table S7).[25] Given its limited availability, we assumed that PET/CT was not used for staging, but studied its use in sensitivity analysis. For patients who underwent MRI of the head and neck as part of screening, this was not repeated during diagnostic workup. Clinical staging was defined by the American Joint Committee on Cancer (AJCC) 7th edition staging system used by Chan et al. to stage screen-detected cases.[26]

We assumed that patients with stage II-IVB disease were treated with concurrent chemoradiotherapy followed by adjuvant chemotherapy, acknowledging clinical equipoise and practice pattern differences in the use of concurrent, concurrent/adjuvant, and induction/concurrent chemoradiotherapy.[27–30] Patients with cT1N0M0 NPC were treated with definitive radiotherapy alone. We extracted country-level data from the IAEA DIRAC database of radiation therapy facilities (as of June 2020).[13] Given widespread availability of intensity-modulated radiotherapy in the 12 included populations, we assumed that all radiotherapy was intensity-modulated and image-guided to a total dose of 70 Gy in 35 daily fractions (Supplementary Table S7).[25] We studied a range of fractionation schemes (30-40 fractions) in sensitivity analysis. While undergoing radiotherapy, patients were evaluated by a radiation oncologist once weekly for side effect management. Multidisciplinary consultation, restaging imaging, endoscopic exam, and tissue confirmation were performed for patients who developed local or distant recurrence after definitive therapy (Supplementary Table S8).

For patients receiving chemotherapy, a separate medical oncology consultation was included, with one follow-up visit per cycle (Supplementary Table S9). For patients with stage II-IVB disease, concurrent chemotherapy was cisplatin 100 mg/m^2 every three weeks, and adjuvant chemotherapy was cisplatin 80mg/m^2 (day 1) with continuous-infusion fluorouracil 1000mg/m^2/d (days 1-4) every four weeks for three cycles.[27] Patients with either recurrent or *de novo* metastatic (stage IVC) disease were treated with chemotherapy alone (cisplatin 80mg/m^2 day 1 with gemcitabine 1000mg/m^2 days 1 and 8, every three weeks).[31] Additionally, we assumed that half of patients who developed a locoregional recurrence underwent palliative 2D/3D conformal radiotherapy to a dose of 30 Gy in 10 fractions (with an associated short-term decrement in health utility), and studied a range of palliative radiotherapy utilization (10-90%) in sensitivity analysis. Although highly-selected patients in high-resource settings are clinically treated with definitive-intent re-irradiation and/or surgical resection, we did not incorporate these salvage therapies into our model due to the small number of patients eligible for these treatments. We furthermore assumed that radiotherapy was not delivered in the *de novo* metastatic setting. Basic labs were drawn and supportive care (antiemetics, hydration) was administered with each cycle of chemotherapy.

Variant-Agnostic and Variant-Informed Screening Strategies

BALF2 Variant-Agnostic Strategies

We studied a set of seven prospectively-evaluated screening strategies and assessed the impact of adding EBV *BALF2* genotyping to triage samples positive for EBV DNA (Supplementary Table S10).[4–6,32–36] These seven strategies were selected due to dependance upon plasma or nasopharyngeal EBV DNA PCR, as residual extracted nucleic acid could be subjected to *BALF2* genotyping. These strategies included combinations of single-antigen serology, plasma or nasopharyngeal EBV DNA PCR, and nasoendoscopy/MRI. We did not study the impact of *BALF2* genotyping on strategies relying only on serology. Screened participants from these trials resided in Zhongshan, Sihui, Wuzhou, and Hong Kong.

We compared seven unique onetime screening strategies against no screening in each studied population. These seven variant-agnostic strategies were:

- A_0: EBV *BamHI-W* DNA plasma PCR [Any Amplification] → Endoscopy
- B_0: EBV *BamHI-W* DNA plasma PCR [Any Amplification] → MRI nasopharynx
- C_0: EBV *BamHI-W* DNA plasma PCR [Any Amplification] → Endoscopy + MRI nasopharynx
- D_0: EBV *BamHI-W* DNA plasma PCR [Any Amplification] → EBV *BamHI-W* DNA plasma PCR [Any Amplification] → Endoscopy
- E_0: EBV *BamHI-W* DNA plasma PCR [Any Amplification] → EBV *BamHI-W* DNA plasma PCR [Any Amplification] → MRI nasopharynx

F_0: EBV *BamHI-W* DNA plasma PCR [Any Amplification] → EBV *BamHI-W* DNA plasma PCR [Any Amplification] → Endoscopy + MRI nasopharynx
G_0: Serum EBV VCA IgA [>1:5] → EBV *BamHI-W* DNA nasopharyngeal swab PCR [Mean+2SD] → Endoscopy

Studies were identified via a prior systematic review of the literature.[3] Identical screening strategies across studies were pooled and weighted by the number of cases and controls using contingency tables.

In Chan et al. (2013), 1,318 men and women age 40-60 underwent plasma EBV *BamHI-W* PCR (any amplification) and serologic testing by ELISA for EBV IgA VCA (ratio >1.0).[35] All patients with a positive initial test by either method underwent nasoendoscopy and a follow-up plasma EBV DNA PCR. We studied the relative performance of plasma EBV DNA PCR → nasoendoscopy (strategy A) and plasma EBV DNA PCR → PCR → nasoendoscopy (strategy D).

In Chan et al. (2017), 20,174 ethnically Chinese men who were 40-62 years of age residing in Hong Kong underwent screening from 2013-2016.[32] Subjects underwent an initial screen for plasma EBV DNA via a quantitative PCR assay amplifying the *BamHI-W* fragment of the EBV genome.[2,37] In subjects with a positive initial screen (any amplification), the PCR was repeated one month later. Those with persistently-positive results (1.5%) underwent endoscopic examination and MRI of the nasopharynx. All screened adults were interviewed annually, and ultimately a total of 35 cases of NPC were detected within one year of screening. Only one of these 35 patients had a negative screen, and presented with stage II NPC within four months of enrollment, corresponding to a sensitivity of 97.1% and specificity of 98.6%. Only 32 unnecessary biopsies were performed among the 20,174 participants (0.2%). We studied scenarios in which all patients were referred for endoscopy and/or MRI after the first positive PCR (strategies A, B, C) and after the second positive PCR (strategies D, E, F). Only 3 of 35 screen-detected patients had negative endoscopic findings with positive MRI findings that prompted a diagnosis of NPC, and therefore we studied triage by endoscopy alone (strategies A, D), MRI alone (strategies B, E), and endoscopy with MRI (strategies C, F). For strategies B and E, patients would need to be referred for endoscopy for examination/biopsy.

Finally, Chen et al. (2015) reported results from a prospective single arm screening study of 22,186 participants.[36] In this study, participants first underwent serologic testing for EBV anti-VCA IgA (>1:5). If this was positive, a nasopharyngeal swab was performed and amplified for EBV DNA by PCR (optimal positive cutoff: mean + 2SD) (strategy G). This study offered similar sensitivity to other studies but had the highest reported specificity (99.95%).

BALF2 Variant-Informed Strategies
For subjects who screen positive for plasma or nasopharyngeal EBV DNA, a subset will have low-risk *BALF2* haplotypes that may prompt discontinuation of further lifetime screening due to the low proportion of endemic NPC cases harboring low-risk haplotypes. This is based on the assumption that haplotypes are stable over one's lifetime (Figure 2).

In the base case, a single lifetime screen was evaluated. In sensitivity analyses, repeated interval screening was also evaluated which increase absolute risk reduction. As the cumulative incidence of subjects with at least one positive EBV PCR increases with interval screening, a growing proportion of the population may be genotyped and excluded from further lifetime screening (Figure S3). This is contingent on the underlying screening strategy (e.g., initial plasma EBV DNA PCR vs. initial anti-VCA IgA ELISA). Accordingly, the impact of variant-informed screening upon referral rates becomes more pronounced with more screening events.

For each of the seven variant-agnostic strategies, we evaluated one additional strategy wherein patients with high-risk *BALF2* haplotypes (C-C-C or C-C-T) proceeded to the next step of screening. Subjects testing positive for plasma/nasopharyngeal EBV DNA but with low-risk *BALF2* haplotypes were not referred for further screening and underwent no further screening in their lifetime. These seven additional variant-informed strategies were:

A_{BALF2}: EBV *BamHI-W* DNA plasma PCR [Any Amplification] + BALF2 PCR [CCC/CCT Haplotype] → Endoscopy
B_{BALF2}: EBV *BamHI-W* DNA plasma PCR [Any Amplification] + BALF2 PCR [CCC/CCT Haplotype] → MRI nasopharynx
C_{BALF2}: EBV *BamHI-W* DNA plasma PCR [Any Amplification] + BALF2 PCR [CCC/CCT Haplotype] → Endoscopy + MRI nasopharynx
D_{BALF2}: EBV *BamHI-W* DNA plasma PCR [Any Amplification] + BALF2 PCR [CCC/CCT Haplotype] → EBV *BamHI-W* DNA plasma PCR [Any Amplification] → Endoscopy
E_{BALF2}: EBV *BamHI-W* DNA plasma PCR [Any Amplification] + BALF2 PCR [CCC/CCT Haplotype] → EBV *BamHI-W* DNA plasma PCR [Any Amplification] → MRI nasopharynx
F_{BALF2}: EBV *BamHI-W* DNA plasma PCR [Any Amplification] + BALF2 PCR [CCC/CCT Haplotype] → EBV *BamHI-W* DNA plasma PCR [Any Amplification] → Endoscopy + MRI
G_{BALF2}: Serum EBV VCA IgA [>1:5] → EBV *BamHI-W* DNA nasopharyngeal swab PCR [Mean+2SD] + BALF2 PCR [CCC/CCT Haplotype] → Endoscopy

We considered several other permutations of *BALF2* genotyping which were not included in this study. For example, strategies referring only the highest-risk haplotype (C-C-T) rather than both the C-C-T/C-C-C haplotypes increases the number of false negatives and decreases the number of false positives. We did not evaluate this triage strategy further, because effective screening sensitivity decreases to approximately 60% without an appreciable increase in cost-effectiveness. We also considered *BALF2* genotyping after the second (rather than first) positive PCR in strategies D-F. This results in identical effective sensitivity and specificity, but increases the number of visits for second phlebotomy while decreasing the number of total genotyping tests required. Both approaches have similar cost-effectiveness, and therefore we studied only triage with *BALF2* PCR at the first positive EBV DNA PCR to prioritize decreasing the number of phlebotomy visits while slightly increasing laboratory costs. An added advantage of upfront genotyping occurs with interval testing: rather than 1.5% of individuals subject to genotyping per screening year, 5.5% are genotyped after the first PCR and 40% of these individuals with low-risk haplotypes could defer further screening in their lifetime.

The proportion of NPC cases and non-NPC controls with each haplotype were derived from meta-analysis of the three previously-conducted association studies in predominantly endemic populations.[4–6] Among the 731 NPC cases in these three studies, 84.7% had the highest-risk haplotype (C-C-T) and 93.0% had either the C-C-C or C-C-T high-risk haplotypes. Among the 826 non-NPC controls in these three studies, 44.3% had the highest-risk haplotype (C-C-T) and 60.5% had either the C-C-C or C-C-T high-risk haplotypes. Relative to variant-agnostic screening, variant-informed screening thereby decreases nasoendoscopy/MRI referral rates by 39.5% with a modest relative decrease in screening sensitivity (7.0%).

We assumed that *BALF2* genotyping would be performed in a separate PCR reaction but with the same extracted nucleic acid from plasma or nasopharyngeal EBV DNA PCR. For nasopharyngeal EBV DNA PCR, the cutoff for a positive screen exceeds the *BALF2* genotyping qPCR 95% LLOD, and we therefore assumed a 100% genotyping success rate. In contrast, a subset of positive plasma specimens would be unable to be genotyped due to low viral load. We therefore assumed that this subset of specimens with amplification failure would be referred for endoscopy and/or MRI as part of usual variant-agnostic screening. In Lam et al., 5/34 screen-detected NPC cases had viral load below the *BALF2* qPCR 95% LLOD (Figure 1).[38] In the base case, the plasma genotyping success rate was therefore set at 85%. A range of 70-100% was studied in sensitivity analyses.

<u>Compliance with Screening</u>
Because screening trials were conducted in differing populations with potential for differing access to healthcare, we used compliance estimates from a single study for the base case (Chan et al. 2017) to avoid introducing selection bias among the screening strategies. In this single-arm prospective trial, compliance with plasma EBV DNA PCR → plasma EBV DNA PCR → nasoendoscopy (strategy D) was 97.1%, which fell to 93.2% with PCR → PCR → nasoendoscopy+MRI (strategy F), as some patients declined or were unable to complete MRI. Therefore, for the base case we assumed all strategies had compliance of 97.1% except for strategy C, which incorporated MRI (93.2%). We assumed that compliance for each screening strategy was uniform across populations given similar or higher HDI relative to China, as screening compliance with other cancer screening programs decreases with decreasing HDI.[16] Because cultural differences and healthcare resources among countries likely have a differential impact upon blood-based NPC screening and cervical cancer screening, we considered a range of country-specific compliance in deterministic and probabilistic sensitivity analysis.

<u>Currency Conversion, Inflation, Discounting, and Purchasing Power Parity</u>
Costs were analyzed from the payer's perspective and presented in 2021 international dollars, which have the same purchasing power parity (PPP) as 2021 United States Dollars. The price of local materials and services in each economy's currency were converted to international dollars via the PPP exchange rate.[39,40] The price of commercially-available laboratory reagents and equipment were obtained from vendors in United States Dollars. All costs were inflated to the year 2021, and all costs and utilities were discounted at 3.0% annually.[41] For the purposes of cost estimation in all countries, we collected each country's total population, population age structure, HDI, and PPP-adjusted Gross Domestic Product (GDP) per capita (Supplementary Table S11).

<u>Costs of Treatment for Nasopharyngeal Carcinoma</u>
Given that fee schedules for individual medical services (clinical visits, endoscopy, imaging, radiotherapy, labs, etc.) are not publicly available for microcosting in each of the studied populations, the price of medical goods and services in each economy were instead estimated using the WHO-CHOICE methodology.[42] This was used in combination with the 2021 United States Medicare fee schedules and pharmaceutical average sales prices. These fee schedules set reimbursement for physician services[43], practice expenses (equipment, supplies, personnel)[43], laboratory testing[44], and pharmaceuticals[45] for patients insured under Medicare. The cost of all unit services involved in the diagnosis and treatment of NPC were obtained from these fee schedules and tabulated to estimate the cost in the United States for NPC diagnosis and a course of definitive radiotherapy (Supplementary Table S7), definitive chemoradiotherapy (Supplementary Tables S7 and S9), and chemotherapy for recurrent or metastatic disease (Supplementary Tables S9).

To convert the cost of diagnosis and treatment in the United States to estimated costs in each included economy, WHO-CHOICE regression models were used.[42] WHO-CHOICE is an initiative of the World Health Organization that seeks to assist countries in setting healthcare priorities, with a variety of tools developed for generalized cost-effectiveness analyses applied to epidemiological subregions.[46] One such tool facilitates estimation of unit costs in the outpatient and inpatient settings for 191 member states based upon a regression model developed from more than 10,000 facility-level observations among these 191 countries.[42] This model incorporates a given economy's GDP per capita (PPP)[47], hospital occupancy rate, average length of hospital stay, outpatient volume, provider/patient ratios, and other data to predict the unit cost of outpatient or inpatient care. Given that radiotherapy and chemotherapy are limited resources in many nations, we assumed that all costs of workup and treatment were incurred in a public urban outpatient referral hospital setting.

Using each economy's GDP per capita (PPP), the cost of an average outpatient visit was calculated using the WHO-CHOICE model. Then, the costs of diagnosis and treatment for NPC in each economy were calculated from the costs of Medicare services in the United States using the ratio of outpatient costs relative to the United States. We previously validated modeled estimates of the cost of MRI, radiotherapy, and chemotherapy using published estimates from three countries with lower-middle income (India), upper-middle income (China), and high income (Republic of China).[3] Given the variability in these proportions, all costs were subsequently studied in sensitivity analysis.

Cost of Screening Strategies

Micro-costing was performed to estimate the total cost of screening with *BamHI-W* plasma EBV DNA PCR, *EBNA-1* or *BamHI-W* nasopharyngeal EBV DNA PCR, single-antigen anti-VCA IgA ELISA, and *BALF2* plasma or nasopharyngeal EBV DNA PCR.[40,48] The methods for conducting these PCR and ELISA assays have been previously described.[40,48–50] We chose to employ micro-costing to account for differences in local personnel and transportation costs despite similar international costs of commercially-available laboratory equipment and reagents.

Sample Transportation Costs

We estimated the cost of transporting each collected sample to a clinical laboratory using the method described by Goldhaber and Goldie.[46,51,52] This method incorporates each region's population, land area, population density, proportion of the population that could be eligible for screening, laboratory worker density, proportion of paved roads in each region, average driving speed, driver wages, fuel costs, and vehicle costs as of 2021.[16,40,48] Data were extracted from United Nations, World Bank, and WHO-CHOICE databases. For simplicity, we assumed a uniform population density to define the land area that a given clinical laboratory would serve. We assumed that a driver would make daily trips in each lab's area to collect samples and deliver them to the nearby central laboratory for analysis. Fuel costs were calculated as the product of fuel efficiency, distance traveled to collect samples, and the cost of fuel. Vehicle and maintenance costs were estimated from WHO-CHOICE and survey data and distributed over a 10-year lifespan. Due to uncertainty in transportation efficiency and driving length, we studied transportation costs from 80-400% of our base case estimate.

Sample Collection and Laboratory Resources

We assumed that 10 minutes of one phlebotomist's time would be required for a blood draw.[40] All blood was drawn into commercially-available standard venipuncture tubes with venipuncture needles. We assumed that 15 minutes of one nurse's time would be required to obtain a nasopharyngeal swab.

We updated prior microcosting models for laboratory assays using the WHO's Laboratory Test Costing Tool (LTCT).[53] The LTCT is designed to assist policymakers, health economists, and laboratory directors to estimate the cost of individual assays using the laboratory financial minute (LFM) methodology. The cost to test a single sample with a given assay incorporates the costs of labor (described below), reagents/consumables, and equipment (acquisition, maintenance, time allocation per assay, amortization).

Standard methods for performing the plasma/nasopharyngeal *BamHI-W/EBNA-1* EBV DNA PCR and anti-VCA IgA ELISA were based upon original scientific reports, standard operating protocols from an academic clinical laboratory, and clinical trials used to develop and validate these tests in large screened populations.[1,2,32,37,54–57] Standard commercially-available reagents and consumables were used for processing samples by laboratory technicians.

We estimated laboratory technician time to perform DNA extraction, PCR, ELISA serum dilution and incubation, and test result documentation based on previously-published data and laboratory standard operating protocols.[40,48] This time was uniform across populations. After centrifugation and plasma storage (five minutes per clinical sample), DNA extraction kits were used to extract EBV DNA from plasma at a rate of 12 samples per hour (five minutes per sample or control/calibrator). After DNA extraction, commercially-available PCR master mix, EBV DNA probes, and EBV DNA primers were combined with extracted DNA in 96-well reaction plates (60 minutes per 96 samples or controls/calibrators). Real-time PCR for each plate was then performed over two hours with appropriate internal controls and calibrators (4 controls, 6 calibrators, 86 patient samples). Five minutes of technician time per sample were budgeted for result

interpretation/documentation. For one batch of 86 clinical samples and 10 controls/calibrators, 1,520 minutes of technician time was required, equivalent to 17.67 LFM per clinical sample. The time and costs to perform *BALF2* PCR were identical to *EBNA-1/BamHI-W* PCR, except that the costs of phlebotomy, sample processing, and nucleic acid extraction were not duplicated.

For the anti-VCA IgA immunoassay, blood was first centrifugated (five minutes per sample). Serum from blood samples was then incubated with commercially-available purified EBV-VCA antigen in 96-well ELISA kits per manufacturer instructions (3 hours per plate, 91 samples per plate, 5 controls/calibrators per plate). Results were then read with an ELISA microplate reader system. Five minutes of technician time per sample were budgeted for result interpretation/documentation. For one batch of 91 clinical samples and 5 controls/calibrators, 1,090 minutes of technician time was required, equivalent to 11.98 LFM per clinical sample.

The cost of reusable equipment and maintenance were distributed over a 5-10 year lifespan. We accounted for an additional 5% reagent/consumables wastage and 20% overhead (applied to all costs). We budgeted an additional five minutes of an administrative assistant's time per sample, and estimated that a clinical pathologist would require one hour to review results from one batch of PCR or ELISA samples.[40,48]

Personnel Costs
Occupation-specific wage data for each economy were obtained from the International Labour Organization, which archive wage data for various occupational sectors in 170 countries.[58] The most recent average annual wages in each economy were obtained from the 2020-2021 Global Wage Report, and were assigned as the wage of a laboratory technician based on close agreement with ISCO-08/3 technician wages in occupation-specific ILO and NBER data. Wages were similarly assigned for nurses (ISCO-08/3 associated professional), phlebotomists (ISCO-08/5 service worker), administrative assistants (ISCO-08/4 clerical support worker), vehicle drivers (ISCO-08/9 machine operator), and clinical pathologists (ISCO-08/2 professional) in each of the included economies. We imputed missing data using linear regression models as a function of PPP-adjusted GDP per capita. Wage data were converted to USD by PPP exchange rates, and then inflated to 2021.[39,41]

We previously validated modeled cost estimates methodology against published costs in lower-middle income, upper-middle income, and high-income countries in a prior study.[3]. Using this microcosting framework, the median proportion of observed-to-modeled cost was 81% (range, 71-91%). In sensitivity analyses, we studied a range of 50-200% of total screening costs, 50-200% of individual component costs (phlebotomy, reagents, labor time, labor wages), and 50-400% of transportation costs.

Screening Nasoendoscopy, Examination, and MRI Costs
The costs of nasoendoscopy and MRI were estimated in each country as a function of PPP-adjusted GDP per capita using the aforementioned WHO-CHOICE models, using United States Medicare Fee Schedules as the reference cost. In addition to the cost of endoscopy itself, we incorporated the cost of a single outpatient visit (as estimated by WHO-CHOICE) to account for a history and physical examination prior to each endoscopy. Complications and incidental findings during endoscopy/MRI were assumed to be negligible.[59]

Model Analysis
For each population and screening strategy, incremental cost-effectiveness ratios (ICER) were calculated as the incremental cost per incremental quality-adjusted life-years per screened subject over a lifetime horizon. Based on WHO-CHOICE guidelines, a willingness to pay (WTP) threshold was set at double the local PPP-adjusted per capita GDP (GDP_{ppp}).[46] To facilitate comparisons across currencies and economies, values were reported as the ICER divided by PPP-adjusted per capita GDP. We also studied lower WTP thresholds of 0.5 and 1.0 QALY/GDP_{ppp}.

Although a subset of participants at medium or high serologic risk were retested in the Ji et al. randomized trial, most prospective screening trials have reported performance with only onetime screening. While onetime screening reduces mortality for the small number of screen-detected prevalent cases, real-world screening programs would employ uniform or adaptive interval screening. With more frequent screening, per-test mortality reduction decreases while absolute mortality reduction increases. Therefore, to supplement the base case analysis of onetime screening, we also studied the impact of screening interval (every 1-5 years) upon absolute mortality reduction and cost-effectiveness.

To assess the robustness of the base case analysis, we performed one-way deterministic and probabilistic sensitivity analyses by varying model parameters in each populations across the 18 screening strategies. The range and distributions for each of these parameters are listed in Supplementary Table S6. We studied the impact of varying the costs of reagents, healthcare services, wages, sample transportation, and discount rate. To study uncertainty in laboratory efficiency, the hours required to perform select tasks (phlebotomy, nasopharyngeal swab, PCR, ELISA, etc.) were varied

by 50-200% around the base case. To separately study total screening costs independent of these components, we varied costs by 50-200% around the base case.

Healthcare utilities were varied by twice the published standard deviation around the base case.[17,60,61] We sampled from the 1,000 randomly-generated sets of calibrated transition probabilities and the Dirichlet stage distributions of NPC. Given higher incidence rates, we performed a separate subset analysis to study the cost-effectiveness of screening only men.

To ascertain the most cost-effective age to offer screening in each population, we varied the age at first screening from 40-60 in five-year increments. We also studied the impact of variable rates of WHO II/III NPC, compliance with screening regimens, number of radiotherapy fractions, and utilization of palliative radiotherapy. We sampled from the 95% confidence intervals for LRR, DM, and OS models to study uncertainty in recurrence rates. Probabilistic sensitivity analysis was performed with 1,000 iterations in each population to study the impact of parameter uncertainty upon cost-effectiveness. We incorporated correlation in health utility uncertainty distributions based on commonsense preferences for those states using a preference order matrix.[62] This study was conducted in accordance with CHEERS reporting standards.[63]

Statistical Analysis
Positive percent agreement (PPA) and negative percent agreement (NPA) were reported with Clopper-Pearson score 95% binomial confidence intervals using NGS as the reference method. The 95% LLOD was calculated using probit regression for each target. Linear regression was used to fit C_t values against nominal concentrations. Odds ratios for high-risk haplotypes (C-C-T and/or C-C-C at positions 162215-162476-163364) were calculated using the common low-risk haplotypes as reference (sum of A-T-C and C-T-C). For EBV-positive NPC cases, the reference group includes all non-NPC controls for each individual study (present cohort, Xu et al., Hui et al., Lam et al.).[4–6] Fisher exact tests were used to calculate *p*-values for SNV and haplotype associations with NPC. For targeted NGS, the *p*-value threshold for statistical significance was adjusted for the number of evaluated positions using the Bonferroni correction ($\alpha = 3.68 \times 10^{-5}$). Analyses were conducted using the *R* statistical software package. This study was reported in accordance STARD and CHEERS guidelines.[63,64]

References

1. Abeynayake J, Johnson R, Libiran P, et al. Commutability of the Epstein-Barr virus WHO international standard across two quantitative PCR methods. *J Clin Microbiol*. 2014;52(10):3802-3804. doi:10.1128/JCM.01676-14

2. Le QT, Zhang Q, Cao H, et al. An international collaboration to harmonize the quantitative plasma Epstein-Barr virus DNA assay for future biomarker-guided trials in nasopharyngeal carcinoma. *Clin Cancer Res Off J Am Assoc Cancer Res*. 2013;19(8):2208-2215. doi:10.1158/1078-0432.CCR-12-3702

3. Miller JA, Le QT, Pinsky BA, Wang H. Cost-Effectiveness of Nasopharyngeal Carcinoma Screening With Epstein-Barr Virus Polymerase Chain Reaction or Serology in High-Incidence Populations Worldwide. *J Natl Cancer Inst*. 2021;113(7):852-862. doi:10.1093/jnci/djaa198

4. Xu M, Yao Y, Chen H, et al. Genome sequencing analysis identifies Epstein-Barr virus subtypes associated with high risk of nasopharyngeal carcinoma. *Nat Genet*. 2019;51(7):1131-1136. doi:10.1038/s41588-019-0436-5

5. Lam WKJ, Ji L, Tse OYO, et al. Sequencing Analysis of Plasma Epstein-Barr Virus DNA Reveals Nasopharyngeal Carcinoma-Associated Single Nucleotide Variant Profiles. *Clin Chem*. 2020;66(4):598-605. doi:10.1093/clinchem/hvaa027

6. Hui KF, Chan TF, Yang W, et al. High risk Epstein-Barr virus variants characterized by distinct polymorphisms in the EBER locus are strongly associated with nasopharyngeal carcinoma. *Int J Cancer*. 2019;144(12):3031-3042. doi:10.1002/ijc.32049

7. Bray F, Colombet M, Mery L, et al. *Cancer Incidence in Five Continents, Vol. XI*. Lyon: International Agency for Research on Cancer; 2017. Accessed January 20, 2019. http://ci5.iarc.fr

8. Forman D, Bray F, Brewstern D, et al. Cancer Incidence in Five Continents, Vol. X. Published 2013. Accessed January 20, 2019. http://ci5.iarc.fr/CI5-X/Default.aspx

9. Sung H, Ferlay J, Siegel RL, et al. Global Cancer Statistics 2020: GLOBOCAN Estimates of Incidence and Mortality Worldwide for 36 Cancers in 185 Countries. *CA Cancer J Clin*. 2021;71(3):209-249. doi:10.3322/caac.21660

10. Taiwan Cancer Registry. Published online 2019. Accessed January 7, 2019. http://tcr.cph.ntu.edu.tw/main.php?Page=N2

11. Cancer Registry - National Registry Of Diseases Office. Accessed January 22, 2022. https://www.nrdo.gov.sg/publications/cancer

12. Hong Kong Cancer Registry: Nasopharyngeal Cancer. Published online 2016. Accessed January 20, 2019. http://www3.ha.org.hk/cancereg/pdf/factsheet/2016/npc_2016.pdf

13. DIrectory of RAdiotherapy Centres (DIRAC). Published April 3, 2019. Accessed July 2, 2020. https://www.iaea.org/resources/databases/dirac

14. Bray F, Ferlay J, Soerjomataram I, Siegel RL, Torre LA, Jemal A. Global cancer statistics 2018: GLOBOCAN estimates of incidence and mortality worldwide for 36 cancers in 185 countries. *CA Cancer J Clin*. 2018;68(6):394-424. doi:10.3322/caac.21492

15. Filipovic-Pierucci A, Zarca K, Wiener M, et al. *Heemod: Markov Models for Health Economic Evaluations*.; 2017. https://CRAN.R-project.org/package=heemod

16. The Global Health Observatory 2017 Update. Published online 2017. Accessed January 10, 2019. https://www.who.int/gho/en/

17. Pow EHN, Kwong DLW, McMillan AS, et al. Xerostomia and quality of life after intensity-modulated radiotherapy vs. conventional radiotherapy for early-stage nasopharyngeal carcinoma: initial report on a randomized controlled clinical trial. *Int J Radiat Oncol Biol Phys*. 2006;66(4):981-991. doi:10.1016/j.ijrobp.2006.06.013

18. Poon DMC, Kam MKM, Johnson D, Mo F, Tong M, Chan ATC. Durability of the parotid-sparing effect of intensity-modulated radiotherapy (IMRT) in early stage nasopharyngeal carcinoma: A 15-year follow-up of a randomized prospective study of IMRT versus two-dimensional radiotherapy. *Head Neck*. 2021;43(6):1711-1720. doi:10.1002/hed.26634

19. Chie WC, Hong RL, Lai CC, Ting LL, Hsu MM. Quality of life in patients of nasopharyngeal carcinoma: validation of the Taiwan Chinese version of the EORTC QLQ-C30 and the EORTC QLQ-H&N35. *Qual Life Res Int J Qual Life Asp Treat Care Rehabil*. 2003;12(1):93-98. doi:10.1023/a:1022070220328

20. Harrington KJ, Ferris RL, Blumenschein G, et al. Nivolumab versus standard, single-agent therapy of investigator's choice in recurrent or metastatic squamous cell carcinoma of the head and neck (CheckMate 141): health-related quality-of-life results from a randomised, phase 3 trial. *Lancet Oncol*. 2017;18(8):1104-1115. doi:10.1016/S1470-2045(17)30421-7

21. Noel CW, Stephens RF, Su JS, et al. Mapping the EORTC QLQ-C30 and QLQ-H&N35, onto EQ-5D-5L and HUI-3 indices in patients with head and neck cancer. *Head Neck*. 2020;42(9):2277-2286. doi:10.1002/hed.26181

22. Gareen IF, Duan F, Greco EM, et al. Impact of lung cancer screening results on participant health-related quality of life and state anxiety in the National Lung Screening Trial. *Cancer*. 2014;120(21):3401-3409. doi:10.1002/cncr.28833

23. Tosteson ANA, Skinner JS, Tosteson TD, et al. The cost effectiveness of surgical versus nonoperative treatment for lumbar disc herniation over two years: evidence from the Spine Patient Outcomes Research Trial (SPORT). *Spine*. 2008;33(19):2108-2115.

24. Loimu V, Mäkitie AA, Bäck LJ, et al. Health-related quality of life of head and neck cancer patients with successful oncological treatment. *Eur Arch Oto-Rhino-Laryngol Off J Eur Fed Oto-Rhino-Laryngol Soc EUFOS Affil Ger Soc Oto-Rhino-Laryngol - Head Neck Surg*. 2015;272(9):2415-2423. doi:10.1007/s00405-014-3169-1

25. NCCN Clinical Practice Guidelines in Oncology. Head and Neck Cancers. Version 1.2019. Published online March 6, 2019. Accessed April 1, 2019. https://www.nccn.org/professionals/physician_gls/pdf/head-and-neck.pdf

26. Edge SB, Compton CC. The American Joint Committee on Cancer: the 7th edition of the AJCC cancer staging manual and the future of TNM. *Ann Surg Oncol*. 2010;17(6):1471-1474. doi:10.1245/s10434-010-0985-4

27. Al-Sarraf M, LeBlanc M, Giri PG, et al. Chemoradiotherapy versus radiotherapy in patients with advanced nasopharyngeal cancer: phase III randomized Intergroup study 0099. *J Clin Oncol Off J Am Soc Clin Oncol*. 1998;16(4):1310-1317. doi:10.1200/JCO.1998.16.4.1310

28. Wee J, Tan EH, Tai BC, et al. Randomized trial of radiotherapy versus concurrent chemoradiotherapy followed by adjuvant chemotherapy in patients with American Joint Committee on Cancer/International Union against cancer stage III and IV nasopharyngeal cancer of the endemic variety. *J Clin Oncol Off J Am Soc Clin Oncol*. 2005;23(27):6730-6738. doi:10.1200/JCO.2005.16.790

29. Blanchard P, Lee A, Marguet S, et al. Chemotherapy and radiotherapy in nasopharyngeal carcinoma: an update of the MAC-NPC meta-analysis. *Lancet Oncol*. 2015;16(6):645-655. doi:10.1016/S1470-2045(15)70126-9

30. Zhang Y, Chen L, Hu GQ, et al. Gemcitabine and Cisplatin Induction Chemotherapy in Nasopharyngeal Carcinoma. *N Engl J Med*. 2019;381(12):1124-1135. doi:10.1056/NEJMoa1905287

31. Zhang L, Huang Y, Hong S, et al. Gemcitabine plus cisplatin versus fluorouracil plus cisplatin in recurrent or metastatic nasopharyngeal carcinoma: a multicentre, randomised, open-label, phase 3 trial. *Lancet Lond Engl*. 2016;388(10054):1883-1892. doi:10.1016/S0140-6736(16)31388-5

32. Chan KCA, Woo JKS, King A, et al. Analysis of Plasma Epstein-Barr Virus DNA to Screen for Nasopharyngeal Cancer. *N Engl J Med*. 2017;377(6):513-522. doi:10.1056/NEJMoa1701717

33. Ji MF, Sheng W, Cheng WM, et al. Incidence and mortality of nasopharyngeal carcinoma: interim analysis of a cluster randomized controlled screening trial (PRO-NPC-001) in southern China. *Ann Oncol*. 2019;30(10):1630-1637. doi:10.1093/annonc/mdz231

34. Liu Z, Ji MF, Huang QH, et al. Two Epstein-Barr virus-related serologic antibody tests in nasopharyngeal carcinoma screening: results from the initial phase of a cluster randomized controlled trial in Southern China. *Am J Epidemiol*. 2013;177(3):242-250. doi:10.1093/aje/kws404

35. Chan KCA, Hung ECW, Woo JKS, et al. Early detection of nasopharyngeal carcinoma by plasma Epstein-Barr virus DNA analysis in a surveillance program. *Cancer*. 2013;119(10):1838-1844. doi:10.1002/cncr.28001

36. Chen Y, Zhao W, Lin L, et al. Nasopharyngeal Epstein-Barr Virus Load: An Efficient Supplementary Method for Population-Based Nasopharyngeal Carcinoma Screening. *PloS One*. 2015;10(7):e0132669. doi:10.1371/journal.pone.0132669

37. Lo YM, Chan LY, Lo KW, et al. Quantitative analysis of cell-free Epstein-Barr virus DNA in plasma of patients with nasopharyngeal carcinoma. *Cancer Res*. 1999;59(6):1188-1191.

38. Lam WKJ, Jiang P, Chan KCA, et al. Sequencing-based counting and size profiling of plasma Epstein–Barr virus DNA enhance population screening of nasopharyngeal carcinoma. *Proc Natl Acad Sci*. 2018;115(22):E5115-E5124. doi:10.1073/pnas.1804184115

39. OECD. OECD Purchasing power parities (PPP) (indicator). Published 2019. Accessed January 20, 2019. http://data.oecd.org/conversion/purchasing-power-parities-ppp.htm

40. Goldie SJ, Gaffikin L, Goldhaber-Fiebert JD, et al. Cost-effectiveness of cervical-cancer screening in five developing countries. *N Engl J Med*. 2005;353(20):2158-2168. doi:10.1056/NEJMsa044278

41. CPI Home : U.S. Bureau of Labor Statistics. Accessed January 20, 2019. https://www.bls.gov/cpi/

42. Stenberg K, Lauer JA, Gkountouras G, Fitzpatrick C, Stanciole A. Econometric estimation of WHO-CHOICE country-specific costs for inpatient and outpatient health service delivery. *Cost Eff Resour Alloc CE*. 2018;16. doi:10.1186/s12962-018-0095-x

43. Centers for Medicare and Medicaid Services. 2019 Medicare Physician Fee Schedule. Published 2019. Accessed January 20, 2019. https://www.cms.gov/Medicare/Medicare-Fee-for-Service-Payment/PhysicianFeeSched/index.html

44. Centers for Medicare and Medicaid Services. 2019 Medicare Clinical Laboratory Fee Schedule. Published 2019. Accessed January 20, 2019. https://www.cms.gov/Medicare/Medicare-Fee-for-Service-Payment/ClinicalLabFeeSched/Clinical-Laboratory-Fee-Schedule-Files-Items/19CLABQ1.html?DLPage=1&DLEntries=10&DLSort=2&DLSortDir=descending

45. Centers for Medicare and Medicaid Services. 2019 Medicare Average Sales Price Drug Pricing Schedule. Published 2019. Accessed January 20, 2019. https://www.cms.gov/Medicare/Medicare-Fee-for-Service-Part-B-Drugs/McrPartBDrugAvgSalesPrice/2019ASPFiles.html

46. World Health Organization (Geneva). Choosing interventions that are cost-effective. Published online 2014. Accessed January 20, 2019. http://www.who.int/choice/en/

47. International Monetary Fund. International Monetary Fund World Economic Outlook. Published online 2018. Accessed January 20, 2019. https://www.imf.org/external/pubs/ft/weo/2018/02/weodata/

48. Goldhaber-Fiebert JD, Goldie SJ. Estimating the cost of cervical cancer screening in five developing countries. *Cost Eff Resour Alloc CE*. 2006;4:13. doi:10.1186/1478-7547-4-13

49. Goldie SJ, Kuhn L, Denny L, Pollack A, Wright TC. Policy analysis of cervical cancer screening strategies in low-resource settings: clinical benefits and cost-effectiveness. *JAMA*. 2001;285(24):3107-3115.

50. Dalvie MA, Sinanovic E, London L, Cairncross E, Solomon A, Adam H. Cost analysis of ELISA, solid-phase extraction, and solid-phase microextraction for the monitoring of pesticides in water. *Environ Res*. 2005;98(1):143-150. doi:10.1016/j.envres.2004.09.002

51. World Bank. World Bank Open Data. Published online 2018. Accessed January 20, 2019. https://data.worldbank.org/

52. United Nations Department of Economic and Social Affairs. World Population Prospects: The 2017 Revision. Published 2017. Accessed January 20, 2019. https://www.un.org/development/desa/publications/world-population-prospects-the-2017-revision.html

53. Laboratory test costing tool-user manual (2019). Accessed January 22, 2022. https://www.euro.who.int/en/health-topics/Health-systems/laboratory-services/publications/laboratory-test-costing-tool-user-manual-2019

54. Chan KH, Gu YL, Ng F, et al. EBV specific antibody-based and DNA-based assays in serologic diagnosis of nasopharyngeal carcinoma. *Int J Cancer*. 2003;105(5):706-709. doi:10.1002/ijc.11130

55. Fachiroh J, Paramita DK, Hariwiyanto B, et al. Single-assay combination of Epstein-Barr Virus (EBV) EBNA1- and viral capsid antigen-p18-derived synthetic peptides for measuring anti-EBV immunoglobulin G (IgG) and IgA antibody levels in sera from nasopharyngeal carcinoma patients: options for field screening. *J Clin Microbiol*. 2006;44(4):1459-1467. doi:10.1128/JCM.44.4.1459-1467.2006

56. Paramita DK, Fachiroh J, Haryana SM, Middeldorp JM. Two-step Epstein-Barr virus immunoglobulin A enzyme-linked immunosorbent assay system for serological screening and confirmation of nasopharyngeal carcinoma. *Clin Vaccine Immunol CVI*. 2009;16(5):706-711. doi:10.1128/CVI.00425-08

57. Zong YS, Sham JS, Ng MH, et al. Immunoglobulin A against viral capsid antigen of Epstein-Barr virus and indirect mirror examination of the nasopharynx in the detection of asymptomatic nasopharyngeal carcinoma. *Cancer*. 1992;69(1):3-7.

58. International Labour Organization. Global Wage Report 2018/19. Published January 1, 2019. Accessed May 29, 2019. https://www.ilo.org/global/research/global-reports/global-wage-report/2018/lang--en/index.htm

59. Lang BHH, Chu KKW, Tsang RKY, Wong KP, Wong BYH. Evaluating the Incidence, Clinical Significance and Predictors for Vocal Cord Palsy and Incidental Laryngopharyngeal Conditions before Elective Thyroidectomy: Is There a Case for Routine Laryngoscopic Examination? *World J Surg*. 2014;38(2):385-391. doi:10.1007/s00268-013-2259-3

60. Kim SH, Jo MW, Kim HJ, Ahn JH. Mapping EORTC QLQ-C30 onto EQ-5D for the assessment of cancer patients. *Health Qual Life Outcomes*. 2012;10:151. doi:10.1186/1477-7525-10-151

61. Truong MT, Zhang Q, Rosenthal DI, et al. Quality of Life and Performance Status From a Substudy Conducted Within a Prospective Phase 3 Randomized Trial of Concurrent Accelerated Radiation Plus Cisplatin With or Without Cetuximab for Locally Advanced Head and Neck Carcinoma: NRG Oncology Radiation Therapy Oncology Group 0522. *Int J Radiat Oncol Biol Phys*. 2017;97(4):687-699. doi:10.1016/j.ijrobp.2016.08.003

62. Goldhaber-Fiebert JD, Jalal HJ. Some Health States Are Better Than Others: Using Health State Rank Order to Improve Probabilistic Analyses. *Med Decis Mak Int J Soc Med Decis Mak*. 2016;36(8):927-940. doi:10.1177/0272989X15605091

63. Husereau D, Drummond M, Petrou S, et al. Consolidated Health Economic Evaluation Reporting Standards (CHEERS)--explanation and elaboration: a report of the ISPOR Health Economic Evaluation Publication Guidelines Good Reporting Practices Task Force. *Value Health J Int Soc Pharmacoeconomics Outcomes Res*. 2013;16(2):231-250. doi:10.1016/j.jval.2013.02.002

64. Bossuyt PM, Reitsma JB, Bruns DE, et al. STARD 2015: An Updated List of Essential Items for Reporting Diagnostic Accuracy Studies. *Radiology*. 2015;277(3):826-832. doi:10.1148/radiol.2015151516

Supplementary Table S1. EBV *BALF2* Genotyping qPCR Primer and Probe Oligonucleotide Sequences and Characteristics

Oligonucleotide	Sequence (5' → 3')	5' Modification	3' Modification	Tm* Match (°C)	Tm* Mismatch (°C)	Sequence Conservation (n=1,050)**
V700_FWD	GCGACCTGCCAGACCT	-	-	63.4	-	1,037 (98.8%)
V700 _REV	CACAGCATCAGCACCTTGGA	-	-	64.6	-	1,042 (99.2%)
V700_WT_FAM	CAGGGCCGGGTGTAC	FAM	$BHQ1_{plus}$	65.2	60.7	822 (78.3%)
V700L_MT_CAL560	CAGGGCCGGTTGTAC	CAL560	$BHQ1_{plus}$	63.4	55.6	219 (20.9%)
I613_FWD	GGTCAGCAGTGAGCGGTAAAAC	-	-	65.3	-	1,036 (98.7%)
I613_REV	CAAGGACCTGGTCAAGAGCTG	-	-	64.3	-	1,042 (99.2%)
I613V_MT_CAL610	CTGGGTGAAGACGGGGCA	CAL610	$BHQ2_{plus}$	71.3	64.3	391 (37.2%)
V317_FWD	TGTTTGCCGACTGTGAGGG	-	-	64.4	-	1,043 (99.3%)
V317_REV	GGCTGGCATTATATCGGTGTAACG	-	-	65.0	-	1,039 (99.0%)
V317M_MT_Q670	AGAGGCCCGTATGGCTG	Q670	$BHQ2_{plus}$	65.2	62.6	307 (29.2%)

Values are presented as number (percent). Tm, melt temperature; FWD, forward; REV, reverse; WT, wild-type; MT, mutant.
*Calculated using IDT OligoAnalyzer for primers using qPCR conditions (DNA, 0.2µM [oligonucleotide], 50mM [Na^+], 3mM [Mg^{2+}],0.8mM [dNTPs]. Calculated using BioSearch RealTimeDesign using BHQplus settings for hydrolysis probes. Mismatch Tm is for wild-type → mutant or mutant → wild-type nucleotide annealing.
**Presence of primer/probe in 1,050 EBV GenBank sequences identified on November 23, 2021 aligning to the EBV BALF2 region of interest (NC_007605.1:162115-163464) with ≥98% coverage.

Supplementary Table S2. EBV *BALF2* Genotyping qPCR dsDNA Control Oligonucleotides

dsDNA Control Fragment	162215 Allele	162476 Allele	163364 Allele	Length	Sequence (5' → 3')
Risk Alleles [wt/mt/mt]*	C	C	T	483	CACCCTGTTCTTGATCTTGATGTTCCTGGGGCACAGCATCAGCACCTTGGACATGCGCACAGGCAGCCGC<br>CGGCCGTACA**C**CCGGCCCTGCAGGGCCGCGTCCAGGTCTGGCAGGTCGCAGGTGGGCTCCCCATGCACCA<br>CCTTGGCCTCCTTGGCCGTGAATTCTCATAGCACATACAGATGGGCAGGGAGATGTCCTGCAGGATGGTC<br>AGCAGTGAGCGGTAAAACAGCTGGGTGAAGA**C**GGGGCAGGCGGGCTGCGCAAAGGGGTTGCACGAGTACT<br>GCATCACGTGGTAGCAGCTCTTGACCAGGTCCTTGTAGGTGAGTAGAGGACGGAATTGGTGGCAAAGATC<br>TGCGTGGACACGTGGGGGGCCAGGCTGGCATTATATCGGTGTAACGCAGCCA**T**ACGGGCCTCTGGACCCT<br>CACAGTCGGCAAACAGGGGCCACGAGTCGTAGTTGAGGCTGGCCGGGGTCTCGTGCGAGGCCT
Non-Risk Alleles [mt/wt/wt]*	A	T	C	483	CACCCTGTTCTTGATCTTGATGTTCCTGGGGCACAGCATCAGCACCTTGGACATGCGCACAGGCAGCCGC<br>CGGCCGTACA**A**CCGGCCCTGCAGGGCCGCGTCCAGGTCTGGCAGGTCGCAGGTGGGCTCCCCATGCACCA<br>CCTTGGCCTCCTTGGCCGTGAATTCTCATAGCACATACAGATGGGCAGGGAGATGTCCTGCAGGATGGTC<br>AGCAGTGAGCGGTAAAACAGCTGGGTGAAGA**T**GGGGCAGGCGGGCTGCGCAAAGGGGTTGCACGAGTACT<br>GCATCACGTGGTAGCAGCTCTTGACCAGGTCCTTGTAGGTGAGTAGAGGACGGAATTGGTGGCAAAGATC<br>TGCGTGGACACGTGGGGGGCCAGGCTGGCATTATATCGGTGTAACGCAGCCA**C**ACGGGCCTCTGGACCCT<br>CACAGTCGGCAAACAGGGGCCACGAGTCGTAGTTGAGGCTGGCCGGGGTCTCGTGCGAGGCCT

wt, wild-type; mt, mutant.
*Denotes wild-type or mutant nucleotide at 162215/162476/163364 positions in EBV reference genome (NCBI RefSeq NC_007605.1).

Supplementary Table S3. EBV *BALF2* Genotyping qPCR Reagents and Concentrations

Reagent	Stock Concentration	Volume (µL)	PCR Reaction Concentration
V700_FWD	3,750 nM	2.0*	300 nM
V700 _REV	3,750 nM	-	300 nM
V700_WT_FAM	625 nM	-	50 nM
V700L_MT_CAL560	1,875 nM	-	150 nM
I613_FWD	3,750 nM	-	300 nM
I613_REV	3,750 nM	-	300 nM
I613V_MT_CAL610	625 nM	-	50 nM
V317_FWD	3,750 nM	-	300 nM
V317_REV	3,750 nM	-	300 nM
V317M_MT_Q670	1,875 nM	-	150 nM
FastStart TaqMan Probe Master Mix**	2X	12.5	1X
Nuclease-free Water	-	0.5	-
Template***	-	10.0	-
**Total**	**-**	**25.0**	**-**

MT, mutant; WT, wild-type; nM, nanomolar.
*2.0µL primer/probe mix diluted in Tris-EDTA (10 mM Tris, 1 mM EDTA) at stock concentrations listed above.
**Catalog Numbers 11732-020 and 11732-088.
***IDT gBlock dsDNA control gene fragments (risk or non-risk alleles), wild-type whole-virus control nucleic acids from EBV-infected B95-8 cell line, or extracted nucleic acids from clinical plasma specimens.

Supplementary Table S4. EBV *BALF2* Genotyping qPCR Interpretation and Haplotypes

PCR Template	Real-Time PCR Result				Quality Control	Interpretation and EBV BALF2 Haplotype				
	**V700 (FAM)**	**V700L (CAL560)**	**I613V (CAL610)**	**V317M (Q670)**		**V700 Wild-Type**	**V700L Mutation**	**I613V Mutation**	**V317M Mutation**	**Haplotype and NPC Risk (162215-162476-163364)**
No Template Control	ndet	ndet	ndet	ndet	NTC QC Passed	Not Detected	Not Detected	Not Detected	Not Detected	No Template Control
Wild-Type B95-8 Control	C_t<38	ndet	ndet	ndet	Wild-Type QC Passed	Detected	Not Detected	Not Detected	Not Detected	C-T-C: Low Risk (Wild-Type)
High-Risk dsDNA Control	C_t<38	ndet	C_t<38	C_t<38	High-Risk QC Passed	Detected	Not Detected	Detected	Detected	C-C-T: High Risk Control
Low-Risk dsDNA Control	ndet	C_t<38	ndet	ndet	Low-Risk QC Passed	Not Detected	Detected	Not Detected	Not Detected	A-T-C: Low Risk Control
Patient Specimens	C_t ≤45	ndet	ndet	ndet	Accept	Detected	Not Detected	Not Detected	Not Detected	C-T-C: Low Risk (Wild-Type)
	C_t ≤45	ndet	C_t ≤45	ndet	Accept	Detected	Not Detected	Detected	Not Detected	C-C-C: High Risk
	C_t ≤45	ndet	C_t ≤45	C_t ≤45	Accept	Detected	Not Detected	Detected	Detected	C-C-T: High Risk
	ndet	C_t ≤45	ndet	ndet	Accept	Not Detected	Detected	Not Detected	Not Detected	A-T-C: Low Risk
	C_t ≤45	ndet	ndet	C_t ≤45	Accept	Detected	Not Detected	Not Detected	Detected	C-T-T: Uncommon Haplotype*
	ndet	C_t ≤45	C_t ≤45	ndet	Accept	Not Detected	Detected	Detected	Not Detected	A-C-C: Uncommon Haplotype*
	ndet	C_t ≤45	ndet	C_t ≤45	Accept	Not Detected	Detected	Not Detected	Detected	A-T-T: Uncommon Haplotype*
	ndet	C_t ≤45	C_t ≤45	C_t ≤45	Accept	Not Detected	Detected	Detected	Detected	A-C-T: Uncommon Haplotype*
	ndet	ndet	ndet	ndet	Repeat PCR	Not Detected	Not Detected	Not Detected	Not Detected	No Amplification
	C_t ≤45	C_t ≤45	Any	Any	Review**	Detected	Detected	Any	Any	Cannot Assign Haplotype**
	ndet	ndet	C_t ≤45	ndet	Review**	Not Detected	Not Detected	Detected	Not Detected	Cannot Assign Haplotype**
	ndet	ndet	ndet	C_t ≤45	Review**	Not Detected	Not Detected	Not Detected	Detected	Cannot Assign Haplotype**
	ndet	ndet	C_t ≤45	C_t ≤45	Review**	Not Detected	Not Detected	Detected	Detected	Cannot Assign Haplotype**

Fixed fluorescence thresholds of 200 relative fluorescence units ([RFU], V700-FAM), 100 RFU (V700L-CAL560), 300 RFU (I613V-CAL610), and 300 RFU (V317M-Q670) are used to determine each target's threshold cycle (C_t).

C_t, cycle threshold; ndet, not detected; NTC, no template control; QC, quality control.

*Uncommon haplotypes: Represent <1% of *BALF2* haplotypes in Xu et al. 2019. Haplotype association with NPC risk unknown.

**Cannot assign EBV BALF2 haplotype. May represent mixed infection if both V700 and V700L are detected. May represent amplification failure or non-A/C nucleotide at V700 position if neither V700/V700L are detected.

Supplementary Table S5. Characteristics of Patients with Genotyped Specimens

Characteristic	All Patients	EBV-Positive NPC Cases	Non-NPC Controls
Patients	179	24	155
Age at First Genotyped Specimen (Years)	42 [18-64]	53 [45-65]	33 [18-63]
Maximum Viral Load (EBNA-1 IU/mL plasma)	612 [<100-882,818]	371 [<100-582,699]	614 [<100-882,818]
Male	105 (59%)	18 (75%)	87 (56%)
EBV-Positive NPC	24 (13%)	24 (100%)	0 (0%)
Solid Organ Transplantation*	71 (40%)	0 (0%)	71 (46%)
Liver	40 (22%)	-	40 (26%)
Heart	35 (20%)	-	35 (23%)
Kidney	30 (17%)	-	30 (19%)
Lung	23 (13%)	-	23 (15%)
Small Intestine	5 (3%)	-	5 (3%)
Pancreas	2 (1%)	-	2 (1%)
Bone Marrow Transplantation	51 (28%)	0 (0%)	51 (33%)
Hematologic Neoplasms	76 (42%)	0 (0%)	76 (49%)
B-Cell Lymphomas/Leukemias	34 (19%)	-	34 (22%)
DLBCL	15 (8%)	-	15 (10%)
B-ALL	12 (7%)	-	12 (8%)
Hodgkin Lymphoma	5 (3%)	-	5 (3%)
Burkitt Lymphoma	1 (1%)	-	1 (1%)
Marginal Zone Lymphoma	1 (1%)	-	1 (1%)
T-Cell Lymphomas/Leukemias	22 (12%)	-	22 (14%)
NKTCL	9 (5%)	-	9 (6%)
Angioimmunoblastic T-Cell NHL	4 (2%)	-	4 (3%)
T-PLL	3 (2%)	-	3 (2%)
T-ALL	3 (2%)	-	3 (2%)
Peripheral T-Cell NHL	2 (1%)	-	2 (1%)
Cutaneous T-Cell Lymphoma	1 (1%)	-	1 (1%)
AML	13 (7%)	-	13 (8%)
MPN/MDS	6 (3%)	-	6 (4%)
Plasma Cell Neoplasm	1 (1%)	-	1 (1%)
Other Diagnoses	19 (11%)	0 (0%)	19 (12%)
Acute EBV Infection (Mononucleosis)	10 (6%)	-	10 (6%)
Non-Transplant-Associated EBV Reactivation	6 (3%)	-	6 (4%)
HLH	3 (2%)	-	3 (2%)
Pharmacologic Immunosuppression	111 (62%)	0 (0%)	111 (72%)
Calcineurin Inhibitors	109 (61%)	-	109 (70%)
Mycophenolate	78 (44%)	-	78 (50%)
mTOR Inhibitors	46 (26%)	-	46 (30%)
Azathioprine	8 (4%)	-	8 (5%)
Methotrexate	7 (4%)	-	7 (5%)
Any CMV Viremia	66 (37%)	0 (0%)	66 (43%)

Values are provided as number (percent) or median (range).
EBV, Epstein-Barr Virus; NPC, nasopharyngeal carcinoma; IU, international units; DLBCL, diffuse large B-cell lymphoma; ALL, acute lymphoblastic leukemia; NKTCL, NK/T-cell lymphoma; NHL, non-Hodgkin lymphoma; PLL, prolymphocytic leukemia; AML, acute myeloid leukemia; MPN/MDS, myeloproliferative neoplasm/myelodysplastic syndrome; HLH, hemophagocytic lymphohistiocytosis; CMV, cytomegalovirus.
*Sum exceeds 71 due to select patients having multi-organ transplantation.

Supplementary Table S6. Modeled Transition Probabilities, Management Assumptions, Health Utilities, and Costs with Parameter Ranges for Deterministic and Probabilistic Sensitivity Analyses

Transition Probability	Base Case	Lower Bound	Upper Bound	Distribution
Health to undetected stage I NPC	age/sex/population-specific (incidence databases)			1,000 calibrated sets
Undetected Stage I NPC to Undetected Stage II-IVC NPC	calibrated from screening trials and SEER detection rates (Miller et al. 2021)			matrix of 1,000 calibrated sets
Undetected Stage I-IVC NPC to Detected Stage I-IVC NPC	calibrated from screening trials and SEER detection rates (Miller et al. 2021)			matrix of 1,000 calibrated sets
Screening Test				
Sensitivity	strategy-specific (Supplementary Table 10)	-0.05	+0.05	binomial
Specificity	strategy-specific (Supplementary Table 10)	-0.02	+0.02	binomial
Compliance	strategy-specific (Supplementary Table 10)	-0.05	+0.05	binomial
High-Risk *BALF2* Haplotype Prevalence	93.0% NPC cases, 60.5% non-NPC controls	20% decrease	20% increase	binomial
Initial Stage Distribution of Undetected NPC				
Stage I	0.349	0.283	0.430	Dirichlet
Stage II	0.381	0.317	0.449	Dirichlet
Stage III	0.230	0.174	0.322	Dirichlet
Stage IVA/B	0.040	0.031	0.075	Dirichlet
Stage IVC	0.000	0.000	0.000	
Stage I-IVA/B NPC to LRR	stage-specific, time-dependent (Supplementary Methods)	Upper 95%CI	Upper 95%CI	sampled from modeled 95%CI
Stage I-IVA/B NPC to DM	stage-specific, time-dependent (Supplementary Methods)	Lower 95%CI	Upper 95%CI	sampled from modeled 95%CI
Stage I-IVC NPC to Death	stage-specific, time-dependent (Supplementary Methods)	Lower 95%CI	Upper 95%CI	sampled from modeled 95%CI
Proportion with WHO II/III Histology	population-specific (Supplementary Table 11)	-0.05	+0.05	beta
Hazard Ratio for LRR, 2D/3DCRT vs. IMRT	2.080	1.820	2.370	normal
Hazard Ratio for OS, 2D/3DCRT vs. IMRT	1.700	1.360	2.120	normal
Screening Age	50.000	40.000	60.000	
**Management Assumptions**				
Radiotherapy modality	IMRT (Supplementary Table 11)	3DCRT	IMRT	
Radiotherapy Fractions	35	30	40	
PET/CT staging	No PET/CT	No PET/CT	PET/CT	
Palliative Radiotherapy Utilization (Recurrent Disease)	0.500	0.100	0.900	beta
**Health State Utilities**				
Perfect health	1.000			
False Positive	1.000	0.900 (1 month)	1.000	beta
Recently-diagnosed NPC prior to treatment (true positive) [months 0-1]	0.910	0.860	0.960	beta
Locoregional recurrence, distant metastasis, or *de novo* metastatic disease	0.620	0.570	0.670	beta
Under treatment with intensity-modulated radiotherapy alone [months 1-3]	0.884	0.834	0.934	beta
Under treatment with 2D/3D radiotherapy alone [months 1-3]	0.872	0.822	0.922	beta
Under treatment with intensity-modulated chemoradiotherapy [months 1-3]	0.833	0.783	0.883	beta
Under treatment with 2D/3D chemoradiotherapy [months 1-3]	0.821	0.771	0.871	beta
Remission after intensity-modulated radiotherapy alone [months 4-12]	0.895	0.845	0.945	beta
Remission after 2D/3D radiotherapy alone [months 4-12]	0.893	0.843	0.943	beta
Remission after intensity-modulated chemoradiotherapy [months 4-12]	0.847	0.797	0.897	beta
Remission after 2D/3D chemoradiotherapy [months 4-12]	0.846	0.796	0.896	beta
Remission after intensity-modulated radiotherapy alone [months 12-24]	0.911	0.861	0.961	beta
Remission after 2D/3D radiotherapy alone [months 12-24]	0.893	0.843	0.943	beta
Remission after intensity-modulated chemoradiotherapy [months 12-24]	0.863	0.813	0.913	beta
Remission after 2D/3D chemoradiotherapy [months 12-24]	0.845	0.795	0.895	beta
Remission after intensity-modulated radiotherapy alone [months 24-96]	0.881	0.831	0.931	beta
Remission after 2D/3D radiotherapy alone [months 24-96]	0.846	0.796	0.896	beta
Remission after intensity-modulated chemoradiotherapy [months 24-96]	0.833	0.783	0.883	beta
Remission after 2D/3D chemoradiotherapy [months 24-96]	0.798	0.748	0.848	beta
Remission after intensity-modulated radiotherapy alone [months 96+]	0.793	0.743	0.843	beta
Remission after 2D/3D radiotherapy alone [months 96+]	0.777	0.727	0.827	beta
Remission after intensity-modulated chemoradiotherapy [months 96+]	0.745	0.695	0.795	beta
Remission after 2D/3D chemoradiotherapy [months 96+]	0.729	0.679	0.779	beta
Death	0.000			
**Costs**				
Total Screening Costs	country-specific (Supplementary Table 11)	0.500	2.000	gamma
Radiotherapy (3DCRT or IMRT, Palliative or Definitive)	country-specific (Supplementary Table 11)	0.500	2.000	gamma
Chemotherapy (Concurrent, Adjuvant, or Palliative)	country-specific (Supplementary Table 11)	0.500	2.000	gamma
Diagnostic Imaging (Workup, Recurrence, MRI Screening)	country-specific (Supplementary Table 11)	0.500	2.000	gamma
PCR/ELISA Reagents	assay-specific (Supplementary Table 11)	0.500	2.000	gamma
Sample Transportation	country-specific (Supplementary Table 11)	0.500	4.000	gamma
Nasoendoscopy	country-specific (Supplementary Table 11)	0.500	2.000	gamma
Wages and Labor Time				
Admin. Assistant	country-specific (Supplementary Table 11)	0.500	2.000	gamma
Phlebotomist	country-specific (Supplementary Table 11)	0.500	2.000	gamma
Laboratory Technician	country-specific (Supplementary Table 11)	0.500	2.000	gamma
Driver (Sample Transportation)	country-specific (Supplementary Table 11)	0.500	2.000	gamma
Clinical Pathologist	country-specific (Supplementary Table 11)	0.500	2.000	gamma
Nursing (Nasopharyngeal Swab)	country-specific (Supplementary Table 11)	0.500	2.000	gamma
**Discount Rate**	0.030	0.000	0.050	beta

NPC, nasopharyngeal carcinoma; LRR, locoregional recurrence; 2D/3DCRT, 2D/3D conformal radiotherapy.

Supplementary Table S7. Cost of Diagnosis, Work-up, and Definitive Radiotherapy in the United States

Services: Diagnosis and Work-up	Units	CPT/HCPCS Code(s)	Unit Cost	Total Costs
MRI nasopharynx with/without contrast	1	70543	$389.06	$389.06
CT head/neck with contrast	1	70491	$204.13	$204.13
CT chest without contrast	1	71250	$145.85	$145.85
Diagnostic nasopharyngolaryngoscopy	1	31575	$131.20	$131.20
Needle biopsy	1	38505	$125.96	$125.96
Tissue examination by pathologist	1	88305	$71.53	$71.53
**Total costs**	**1**			**$1,067.73**
**Services: Definitive Intensity-Modulated Radiotherapy**	**Units**	**CPT/HCPCS Code(s)**	**Unit Cost**	**Total Costs**
Physician consult	1	99205	$224.36	$224.36
Clinical treatment Plan	1	77263	$169.93	$169.93
Simulation	1	77290	$501.41	$501.41
Thermoplastic mask	1	77334	$128.06	$128.06
Isodose plan	1	77307	$128.06	$128.06
IMRT planning	1	77301	$1,935.17	$1,935.17
Multileaf collimator	1	77338	$480.48	$480.48
Dosimetry	1	77331	$65.95	$65.95
Daily image guidance	35	G6002	$77.11	$2,698.85
IMRT treatment delivery	35	G6015	$385.57	$13,494.95
Physics consult	1	77370	$130.85	$130.85
Weekly physics quality assurance	7	77336	$82.70	$578.90
Weekly treatment management	7	77427	$191.91	$1,343.37
**Total Costs**				**$21,880.34**

CPT, current procedural terminology; HCPCS, Healthcare Common Procedure Coding System; MRI, magnetic resonance imaging; CT, computed tomography; IMRT, intensity-modulated radiotherapy. All costs are reported in 2021 United States Dollars.

Supplementary Table S8. Cost of Restaging for Local Recurrence or Distant Metastasis

Services	CPT/HCPCS Code(s)	Unit Cost	Total Costs
History and physical (three multi-disciplinary consults)	99205	$224.36	$673.08
Diagnostic nasopharyngolaryngoscopy	31575	$131.20	$131.20
CT chest, abdomen, pelvis with IV contrast	71260, 74177	$523.74	$523.74
Needle biopsy	38505	$125.96	$125.96
Tissue examination by pathologist	88305	$71.53	$71.53
**Total restaging costs**			**$1,525.51**

CPT, current procedural terminology; HCPCS, Healthcare Common Procedure Coding System; CT, computed tomography.
Data are from Medicare Physician Fee Schedule 2021.[65] All costs are reported in 2021 United States Dollars.

Supplementary Table S9. Cost of Chemotherapy and Supportive Care in the United States

Chemotherapy Regimen	CPT/ HCPCS Code(s)	Unit Cost	Unit Dosage (mg)	Cycle Dosage* (mg)	Agent Cost Per Cycle	Supportive Medications and Laboratory Costs	Infusion Costs Per Cycle	Total Costs Per Course
**Concurrent Chemotherapy**								
Physician consult, once	99205	$224.36						$224.36
Follow-up visit, once per cycle	99213	$92.47						$277.41
Cisplatin 100mg/m^2, concurrent, every three weeks	J9060, 96409	$1.89	10	200	$37.00		$113.40	$453.60
Ondansetron 8mg PO, every six hours for one week	Q0162	$0.02	1	224		$3.81		$11.42
Prochlorperazine 10mg PO, every six hours for one week	Q0164	$0.30	5	280		$17.02		$51.07
Dexamethasone 8mg PO, once per cycle	J8540	$0.10	0.25	8		$3.04		$9.12
Normal saline 1L IV, once per cycle	J7030, 96360	$2.79	1000	1000		$2.79	$36.29	$117.24
Complete blood count, per cycle	85025	$7.77				$7.77		$23.31
Complete metabolic panel, per cycle	80053	$10.56				$10.56		$31.68
**Total Costs per Course (3 Cycles)**								**$1,199.22**
**Adjuvant Chemotherapy**								
Follow-up visit, once per cycle	99213	$92.47						$277.41
Cisplatin 80mg/m^2 d1, adjuvant, every four weeks	J9060, 96409	$1.89	10	200	$37.80		$113.40	$453.60
5-Fluorouracil 1000mg/m^2 d1-4, adjuvant, every four weeks	J9190, 96416	$1.79	500	8000	$28.64		$147.25	$527.67
Ondansetron 8mg PO, every six hours for one week	Q0162	$0.02	1	224		$3.81		$11.42
Prochlorperazine 10mg PO, every six hours for one week	Q0164	$0.30	5	280		$17.02		$51.07
Dexamethasone 8mg PO, once per cycle	J8540	$0.10	0.25	8		$3.20		$9.60
Normal Saline 1L IV, once per cycle	J7030, 96360	$2.79	1000	1000		$2.79	$36.29	$117.24
Complete blood count, per cycle	85025	$7.77				$7.77		$23.31
Complete metabolic panel, per cycle	80053	$10.56				$10.56		$31.68
**Total Costs per Course (3 Cycles)**								**$1,503.01**
**Indefinite Chemotherapy for Metastatic Disease**								
Physician consult, once	99205	$224.36						$224.36
Follow-up visit, once per cycle	99213	$92.47						$554.82
Gemcitabine 1000mg/m^2 d1,8, metastatic, every three weeks	J9201, 96409	$4.14	200	4000	$82.86		$226.80	$1,857.96
Cisplatin 80mg/m^2 d1, metastatic, every three weeks	J9060, 96409	$1.89	10	200	$37.80		$113.40	$907.20
Ondansetron 8mg PO, every six hours for one week	Q0162	$0.02	1	224		$3.81		$22.85
Prochlorperazine 10mg PO, every six hours for one week	Q0164	$0.30	5	280		$17.02		$102.14
Dexamethasone 8mg PO, once per cycle	J8540	$0.10	0.25	8		$3.20		$19.20
Normal saline 1L IV, once per cycle	J7030, 96360	$2.79	1000	1000		$2.79	$36.29	$234.48
Complete blood count, per cycle	85025	$7.77				$7.77		$46.62
Complete metabolic panel, per cycle	80053	$10.56				$10.56		$63.36
CT chest, abdomen, pelvis with contrast, every three cycles	71260, 74177	$523.74						$1,047.48
**Total Costs per Course (6 Cycles)**								**$5,080.47**

CPT, current procedural terminology; HCPCS, Healthcare Common Procedure Coding System; PO, oral. Data are from 2021 Medicare Physician Fee Schedule, Laboratory Fee Schedule, and Drug Average Sales Price.[44,45,65] All costs are reported in 2021 United States Dollars. [a]Based on 2.0 m^2 body surface area adult. Palliative chemotherapy is administered for six cycles every three weeks.

Supplementary Table S10. Performance Characteristics and Resource Utilization for Variant-Agnostic and *BALF2* Variant-Informed NPC Screening Strategies. We evaluated seven variant-agnostic and seven corresponding variant-informed screening strategies, which were each compared with no screening. Variant-informed screening increased PPV by a median of 46% (range, 26-51%) and decreased absolute screening sensitivity by 7%. For example, the strategy reported by Chan et al. (strategy F_0, tandem plasma EBV *BamHI-W* DNA followed by MRI and endoscopy) had screening sensitivity and PPV of 97.1% and 11.0%, which changed to 90.4% and 16.0% after triaging the first positive PCR with *BALF2* genotyping. For this identical screened population of 20,174 subjects, this would amount to approximately 2.4 missed NPC cases and 108.5 fewer false-positives.

Strategy ID	Screening Strategy	Person-Years*	Prevalent Cases*	Resource Utilization Per Screened Subject**							Sensitivity	Specificity	PPV	Citation(s)*
				Plasma BamHI-W PCR	NP PCR	ELISA	MRI	Exam + Endoscopy	BALF2 PCR	Visits Per Subject				
A_0	Plasma EBV BamHI-W DNA PCR→<br>Endoscopy	21,492	38	1	0	0	0	0.0555	0	1.0555	89.47%	94.67%	2.89%	[32,35]
A_{BALF2}	Plasma EBV BamHI-W DNA PCR+BALF2 PCR→<br>Endoscopy	-	-	1	0	0	0	0.0338	1	1.0338	82.39%	96.76%	4.23%	[4–6,32,35]
B_0	Plasma EBV BamHI-W DNA PCR→<br>MRI nasopharynx	20,174	35	1	0	0	0.0551	0.0206	0	1.084	97.14%	94.65%	3.06%	[32]
B_{BALF2}	Plasma EBV BamHI-W DNA PCR+BALF2 PCR→<br>MRI nasopharynx	-	-	1	0	0	0.0339	0.0127	1	1.0394	90.37%	96.76%	4.62%	[4–6,32]
C_0	Plasma EBV BamHI-W DNA PCR→<br>Endoscopy+MRI nasopharynx	20,174	35	1	0	0	0.0551	0.0551	0	1.110	97.14%	94.65%	3.06%	[32]
C_{BALF2}	Plasma EBV BamHI-W DNA PCR+BALF2 PCR→<br>Endoscopy+MRI nasopharynx	-	-	1	0	0	0.0339	0.0339	1	1.0678	90.37%	96.76%	4.62%	[4–6,32]
D_0	Plasma EBV BamHI-W DNA PCR→<br>Plasma EBV BamHI-W DNA PCR→<br>Endoscopy	21,492	38	1.0555	0	0	0	0.0153	0	1.0708	89.47%	98.63%	10.43%	[32,35]
D_{BALF2}	Plasma EBV BamHI-W DNA PCR+BALF2 PCR→<br>Plasma EBV BamHI-W DNA PCR→<br>Endoscopy	-	-	1.0334	0	0	0	0.0097	1	1.0430	82.39%	99.17%	14.77%	[4–6,32,35]
E_0	Plasma EBV BamHI-W DNA PCR→<br>Plasma EBV BamHI-W DNA PCR→<br>MRI nasopharynx	20,174	35	1.0551	0	0	0.0153	0.0057	0	1.0795	97.14%	98.63%	11.00%	[32]
E_{BALF2}	Plasma EBV BamHI-W DNA PCR+BALF2 PCR→<br>Plasma EBV BamHI-W DNA PCR→<br>MRI nasopharynx	-	-	1.0334	0	0	0.0098	0.0037	1	1.0457	90.37%	99.17%	15.97%	[4–6,32]
F_0	Plasma EBV BamHI-W DNA PCR→<br>Plasma EBV BamHI-W DNA PCR→<br>Endoscopy+MRI nasopharynx	20,174	35	1.0551	0	0	0.0153	0.0153	0	1.0858	97.14%	98.63%	11.00%	[32]
F_{BALF2}	Plasma EBV BamHI-W DNA PCR+BALF2 PCR→<br>Plasma EBV BamHI-W DNA PCR→<br>Endoscopy+MRI nasopharynx	-	-	1.0334	0	0	0.0098	0.00982	1	1.0530	90.37%	99.17%	15.97%	[4–6,32]
G_0	Serum EBV VCA IgA [>1:5]→<br>Nasopharyngeal EBV BamHI-W DNA PCR [mean+2SD]→<br>Endoscopy	22,186	8	0	0.0482	1	0	0.0009	0	1.0491	87.50%	99.95%	41.18%	[36]
G_{BALF2}	Serum EBV VCA IgA [>1:5]→<br>Nasopharyngeal EBV BamHI-W DNA PCR [mean+2SD]+BALF2 PCR→<br>Endoscopy	-	-	0	0.0482	1	0	0.0007	0.0482	1.0489	81.40%	99.97%	51.82%	[4–6,36]

NPC, nasopharyngeal carcinoma; PCR, polymerase chain reaction; ELISA, enzyme-linked immunosorbent assay; MRI, magnetic resonance imaging; NP, nasopharyngeal; PPV, positive predictive value.

*Screening performance characteristics and resource utilization for variant-agnostic (A_0-I_0) screening strategies are derived from four prospective screening trials.[32,35,36,66] For strategies A_0 and D_0, performance and resource utilization was pooled between Chan et al. 2013 and Chan et al. 2017. For *BALF2* variant-informed screening strategies, high-risk and low-risk haplotype distributions derived from a meta-analysis of Xu et al. 2019, Hui et al. 2019, and Lam et al. 2020 are used to triage plasma/nasopharyngeal specimens positive for EBV DNA. Person-years refers to the number of screening person-years. Prevalent cases refers to the number of true positives and false negatives within one year of screening. For cited studies above, full details regarding screened population, inclusion criteria, screening years, prevalence estimates, stage distributions, and contingency tables may be found in original publications.

**Average resource utilization per screening subject. For example, 5.5% of individuals screen positive by plasma *BamHI-W* PCR and undergo a second PCR for strategies D_0, E_0, F_0, resulting in an average of 1.055 plasma PCR per screened subject. Visits per subject refers to the average number of unique in-person visits per screened subject. This is equal to the sum of plasma PCR, NP PCR, ELISA, MRI, and exam+endoscopy. *BALF2* qPCR is performed from residual extracted nucleic acids, and does not require an additional visit for specimen collection.

Supplementary Table S11. Country-Level Economic and Demographic Data, Including Screening Costs, Rates of WHO Type II/III Histology, and IMRT Availability. Although the cost of reagents/consumables was fixed across populations, the cost to perform PCR/ELISA differed based upon variable personnel and transportation costs. The median estimated cost to perform plasma *BamHI-W* PCR was $36.34 (range, $27.47-40.31), while the median cost to perform anti-VCA IgA ELISA was $20.14 (range, $13.12-23.30). After phlebotomy, sample transportation, and nucleic acid extraction, the median additional cost to perform *BALF2* PCR was $19.97 (range, $12.85-23.05). In these five high and upper-middle income economies (southern China, Hong Kong SAR, Macao SAR, Republic of China, Singapore), total screening costs were approximately 35-65% reagents/consumables, 20-45% personnel costs, and 5-20% endoscopy/MRI costs.

See file, "Supplementary Table S11 - Country-Level Data.xlsx"

Supplementary Table S12. Analytical Performance: Multiplex *BALF2* Genotyping qPCR Lower Limit of Detection in Replicates of 20

dsDNA Control Concentration	Number of Detected Replicates and 95% Lower Limit of Detection			
	**V700 (FAM)**	**V700L (CAL560)**	**I613V (CAL610)**	**V317M (Q670)**
0.1 copies/µL template	13/20	12/20	12/20	12/20
0.5 copies/µL template	20/20	20/20	20/20	20/20
1.0 copies/µL template	20/20	20/20	20/20	20/20
5.0 copies/µL template	20/20	20/20	20/20	20/20
95% LLOD (95% CI) - copies/µL template	0.19 (0.13-0.25)	0.20 (0.14-0.26)	0.20 (0.14-0.26)	0.20 (0.14-0.26)
95% LLOD (95% CI) - copies/reaction*	1.9 (1.3-2.5)	2.0 (1.4-2.6)	2.0 (1.4-2.6)	2.0 (1.4-2.6)
95% LLOD (95% CI) - copies/mL plasma*	11.4 (7.8-15.0)	12.0 (8.4-15.6)	12.0 (8.4-15.6)	12.0 (8.4-15.6)

LLOD, lower limit of detection; CI, confidence interval.
*10 µL nucleic acid template included per 25 µL reaction. LLOD in plasma calculated by extrapolating from nucleic acid extraction protocol: 1000 µL plasma extracted into 60 µL elution buffer (AVE), with 10 µL template in each 25 µL reaction (6X ratio from copies/reaction to copies/mL plasma).

Supplementary Table S13. Analytical Performance: Multiplex *BALF2* Genotyping qPCR Linearity in Replicates of Three Across Six Orders of Magnitude

dsDNA Control Concentration	Replicate C_t Values				Coefficient of Variation (%)*			
	**V700 (FAM)**	**V700L (CAL560)**	**I613V (CAL610)**	**V317M (Q670)**	**V700 (FAM)**	**V700L (CAL560)**	**I613V (CAL610)**	**V317M (Q670)**
10^0 copies/µL template	40.12, 37.56, 38.58	36.37, 36.39, 36.91	38.11, 36.73, 37.57	39.08, 37.70, 39.05	220.72%	38.28%	252.50%	224.00%
10^1 copies/µL template	35.48, 35.23, 35.22	35.04, 34.48, 34.09	34.57, 34.61, 34.66	36.04, 35.78, 35.76	4.20%	17.09%	1.52%	4.93%
10^2 copies/µL template	32.06, 31.39, 31.43	30.56, 30.71, 30.64	31.10, 30.73, 30.96	32.44, 32.09, 32.20	5.18%	1.10%	2.72%	2.67%
10^3 copies/µL template	28.66, 27.99, 28.03	27.08, 27.10, 27.08	27.51, 27.41, 27.50	29.02, 28.75, 28.77	3.55%	0.14%	0.52%	1.51%
10^4 copies/µL template	24.57, 24.28, 24.12	23.12, 23.26, 23.20	23.73, 23.66, 23.53	25.14, 25.03, 24.95	1.57%	0.51%	0.70%	0.69%
10^5 copies/µL template	21.58, 21.21, 21.09	19.83, 19.98, 19.90	21.00, 20.65, 20.56	21.98, 22.08, 22.00	1.44%	0.43%	1.36%	0.33%
R^2	0.9926	0.9927	0.9963	0.9958	-	-	-	

C_t, cycle threshold.
*Based on linear regression standard curves: V700-FAM: copies/µL template = 10^(10.910-0.281*C_t); V700L-CAL560: copies/µL template = 10^(10.742-0.288*C_t); I613V-CAL610: copies/µL template = 10^(10.966-0.291*C_t); V317M-Q670: copies/µL template = 10^(11.431-0.293*C_t).

Supplementary Table S14. Analytical Performance: Multiplex EBV *BALF2* Genotyping qPCR Minor Allele Frequency Detection in Replicates of Three

Risk Allele Frequency (V700L, I613V, V317M)*	Replicate C_t Values				Replicate Measured Concentrations (copies/µL template)**			
	**V700 (FAM)**	**V700L (CAL560)**	**I613V (CAL610)**	**V317M (Q670)**	**V700 (FAM)**	**V700L (CAL560)**	**I613V (CAL610)**	**V317M (Q670)**
0%	ndet, ndet, ndet	30.56, 30.71, 30.64	ndet, ndet, ndet	ndet, ndet, ndet	0.00, 0.00, 0.00	89.46, 81.11, 84.78	0.00, 0.00, 0.00	0.00, 0.00, 0.00
1%	ndet, ndet, ndet	30.48, 30.23, 30.5	ndet, ndet, ndet	ndet, ndet, ndet	0.00, 0.00, 0.00	94.47, 111.66, 93.28	0.00, 0.00, 0.00	0.00, 0.00, 0.00
5%	ndet, ndet, 44.11	30.19, 30.08, 29.95	ndet, ndet, ndet	38.25, 39.51, 38.25	0.00, 0.00, 0.03	114.74, 122.98, 134.18	0.00, 0.00, 0.00	1.62, 0.69, 1.62
10%	40.33, 39.96, 38.88	30.1, 30.05, 29.88	43.73, 43.75, 44.79	37.14, 36.93, 36.29	0.37, 0.48, 0.95	121.79, 126.02, 140.27	0.02, 0.02, 0.01	3.42, 3.95, 6.08
20%	35.06, 34.89, 34.38	30.42, 30.44, 30.35	34.5, 34.73, 34.76	34.5, 34.83, 34.54	11.31, 12.69, 17.58	98.57, 97.03, 103.32	8.76, 7.53, 7.36	20.31, 16.27, 19.80
30%	33.93, 33.82, 33.48	30.81, 30.7, 30.61	33.43, 33.57, 33.26	33.75, 33.93, 33.82	23.61, 25.27, 31.47	76.16, 81.80, 86.68	18.00, 16.39, 20.12	33.75, 29.84, 32.16
40%	32.93, 33.13, 32.64	31.01, 31.11, 31.05	32.42, 32.58, 32.36	33.26, 33.4, 33.04	44.94, 39.62, 54.21	66.74, 62.26, 64.74	35.37, 31.77, 36.66	47.12, 42.96, 54.47
50%	32.44, 32.55, 32.41	31.41, 31.65, 31.51	32.02, 32.09, 31.87	32.97, 32.99, 32.94	61.99, 57.69, 63.05	51.09, 43.60, 47.76	46.14, 44.12, 50.91	57.29, 56.39, 58.53
60%	32.27, 32.15, 32.1	31.87, 32.03, 31.8	31.64, 31.53, 31.51	32.76, 32.64, 32.59	69.23, 74.47, 76.99	37.66, 33.83, 39.50	59.54, 64.08, 64.89	65.99, 71.77, 74.09
70%	32.03, 32.07, 32.01	32.98, 32.94, 33.03	31.19, 31.33, 31.25	32.48, 32.55, 32.54	80.61, 78.47, 81.79	18.07, 18.52, 17.51	80.27, 73.35, 77.17	79.60, 75.98, 76.64
80%	31.36, 31.37, 31.31	33.35, 33.49, 33.6	30.87, 30.87, 30.81	32.23, 32.04, 31.96	124.52, 123.62, 128.33	14.15, 12.89, 11.99	99.20, 99.45, 103.57	94.56, 107.20, 113.62
90%	31.01, 31.02, 31.07	37.16, 37.12, 40.85	30.34, 30.57, 30.52	31.63, 31.73, 31.71	155.83, 155.28, 149.77	1.14, 1.16, 0.10	142.22, 121.60, 125.75	141.30, 132.71, 134.40
95%	31.04, 31.08, 31.05	ndet, ndet, 40.85	30.48, 30.41, 30.41	31.73, 31.7, 31.66	152.77, 148.79, 152.24	0.00, 0.00, 0.10	129.05, 135.25, 134.99	132.68, 134.79, 138.94
99%	31.03, 31.1, 31.26	ndet, ndet, ndet	30.32, 30.38, 30.49	31.66, 31.61, 31.73	153.79, 147.16, 133.02	0.00, 0.00, 0.00	143.72, 138.26, 128.52	138.61, 143.36, 132.68
100%	32.06, 31.39, 31.43	ndet, ndet, ndet	31.1, 30.73, 30.96	32.44, 32.09, 32.2	79.15, 121.71, 118.49	0.00, 0.00, 0.00	85.34, 109.20, 93.97	81.69, 103.47, 96.21

C_t, cycle threshold.
*Total template concentration (dsDNA risk control and dsDNA non-risk control) is fixed at 100 copies/µL, and the proportion of each control is varied from 0-100% of the risk allele.
**Based on standard curves: V700-FAM: copies/µL template = $10^{(10.910-0.281*C_t)}$; V700L-CAL560: copies/µL template = $10^{(10.742-0.288*C_t)}$; I613V-CAL610: copies/µL template = $10^{(10.966-0.291*C_t)}$; V317M-Q670: copies/µL template = $10^{(11.431-0.293*C_t)}$.

Supplementary Table S15. EBV *BALF2* Genotyping qPCR Validation with Targeted Next-Generation Sequencing

BALF2 qPCR	Targeted *BALF2* Next Generation Sequencing							
	V700		V700L		I613V		V317M	
	Detected	Not Detected	Detected	Not Detected	Detected	Not Detected	Detected	Not Detected
**Detected**	129	0	23	0	23	0	9	0
**Not Detected**	0	23	0	129	1	128	0	143
**PPA (95% CI)***	100% (97-100%)		100% (85-100%)		100% (85-100%)		100% (66-100%)	
**NPA (95% CI)***	100% (85-100%)		100% (97-100%)		99% (96-100%)		100% (97-100%)	

PPA, positive percent agreement; NPA, negative percent agreement; CI, confidence interval.
*BALF2 qPCR relative to next-generation sequencing.

Supplementary Table S16. EBV *BALF2* Haplotype Distributions and Association with NPC

Cohort	EBV BALF2 Haplotype (162215-162476-166364)					Odds Ratio (95% CI): NPC vs. Non-NPC		
	**Low Risk: A-T-C**	**Low Risk: C-T-C**	**High Risk: C-C-C**	**High Risk: C-C-T**	**Other**	**High Risk: C-C-C**	**High Risk: C-C-T**	**High Risk: C-C-C or C-C-T**
EBV-Positive NPC Cases	2 (8.3%)	4 (16.7%)	3 (12.5%)	15 (62.5%)	0 (0%)	7.9 (1.7-37.1)	178.8 (33.1-965.3)	39.0 (12.9-118.2)
Non-NPC Controls	17 (11.0%)	126 (81.3%)	9 (5.8%)	2 (1.3%)	1 (0.6%)*	-	-	-
Solid Organ Transplant	5 (7.0%)	63 (88.7%)	3 (4.3%)	0 (0.0%)	0 (0.0%)	-	-	-
Bone Marrow Transplant	8 (15.7%)	37 (72.5%)	4 (7.8%)	1 (2.0%)	1 (2.0%)	-	-	-
Hematologic Neoplasms	10 (13.2%)	60 (78.9%)	4 (5.3%)	1 (1.3%)	1 (1.3%)	0.8 (0.2-3.2)	1.0 (0.1-17.0)	0.9 (0.3-3.0)
B-Cell Lymphomas / Leukemia	4 (11.8%)	26 (76.5%)	3 (8.8%)	1 (2.9%)	0 (0.0%)	1.9 (0.4-8.0)	3.8 (0.2-62.0)	2.2 (0.6-7.8)
B-Cell NHL	1 (5.9%)	13 (76.5%)	2 (11.8%)	1 (5.9%)	0 (0.0%)	2.6 (0.5-13.9)	9.2 (0.5-155.5)	3.5 (0.8-14.5)
B-ALL	2 (16.7%)	9 (75.0%)	1 (8.3%)	0 (0.0%)	0 (0.0%)	1.5 (0.2-13.1)	-	1.2 (0.1-10.3)
Hodgkin Lymphoma	1 (20%)	4 (80%)	0 (0%)	0 (0.0%)	0 (0.0%)	-	-	-
T-Cell Lymphoma/Leukemia	5 (22.8%)	15 (68.2%)	1 (4.5%)	0 (0.0%)	1 (4.5%)	0.8 (0.1-6.5)	-	0.6 (0.1-5.1)
NKTCL	4 (44.4%)	5 (55.6%)	0 (0.0%)	0 (0.0%)	0 (0.0%)	-	-	-
T-Cell Lymphomas / Leukemias	1 (7.7%)	10 (76.9%)	1 (7.7%)	0 (0.0%)	1 (7.7%)	1.5 (0.2-13.1)	-	1.2 (0.1-10.3)
AML	1 (7.7%)	12 (92.3%)	0 (0.0%)	0 (0.0%)	0 (0.0%)	-	-	-
MPN/MDS	0 (0.0%)	6 (100.0%)	0 (0.0%)	0 (0.0%)	0 (0.0%)	-	-	-
Plasma Cell Neoplasm	0 (0.0%)	1 (100.0%)	0 (0.0%)	0 (0.0%)	0 (0.0%)	-	-	-
Acute EBV Infection or Non-Transplant EBV Reactivation	3 (15.8%)	13 (68.4%)	2 (10.5%)	1 (5.3%)	0 (0.0%)	2.3 (0.4-11.9)	7.9 (0.5-133.2)	3.0 (0.7-12.4)
Other Published Cohorts						-	-	-
Xu et al. 2019 EBV-Positive NPC Cases	25 (3.9%)	13 (2.0%)	57 (8.9%)	539 (84.4%)	5 (0.8%)	3.0 (1.9-4.8)	11.4 (7.9-16.6)	9.0 (6.3-13.0)
Xu et al. 2019 Non-NPC Controls	171 (26.2%)	65 (10.0%)	118 (18.1%)	293 (44.9%)	5 (0.8%)	-	-	-
Hui et al. 2019 EBV-Positive NPC Cases	4 (6.5%)	1 (1.6%)	2 (3.2%)	55 (88.7%)	0 (0.0%)	2.0 (0.4-11.4)	13.3 (5.0-35.4)	11.1 (4.2-29.3)
Hui et al. 2019 Non-NPC Controls	53 (37.3%)	17 (12.0%)	14 (9.9%)	58 (40.8%)	0 (0.0%)	-	-	-
Lam et al. 2020 EBV-Positive NPC Cases	2 (6.7%)	1 (3.3%)	2 (6.7%)	25 (83.3%)	0 (0.0%)	5.0 (0.5-50.8)	8.3 (2.1-33.6)	7.9 (2.0-31.6)
Lam et al. 2020 Non-NPC Controls	12 (37.4%)	3 (9.4%)	2 (6.3%)	15 (46.9%)	0 (0.0%)	-	-	-
Xu, Hui, and Lam Aggregated EBV-Positive Cases	31 (4.2%)	15 (2.1%)	61 (8.3%)	619 (84.7%)	5 (0.7%)	3.2 (2.1-4.9)	11.8 (8.4-16.5)	9.5 (6.8-13.2)
Xu, Hui, and Lam Aggregated Non-NPC Controls	236 (28.6%)	85 (10.3%)	134 (16.2%)	366 (44.3%)	5 (0.6%)	-	-	-
All EBV-Positive NPC Cases	33 (4.3%)	19 (2.5%)	64 (8.5%)	634 (84.0%)	5 (0.7%)	4.0 (2.6-6.0)	15.4 (11.2-21.0)	12.2 (9.0-16.6)
All Non-NPC Controls	253 (25.8%)	211 (21.5%)	143 (14.6%)	368 (37.5%)	6 (0.6%)	-	-	-

Values are presented as number (percent) or odds ratio (95% confidence interval [CI]). *Odds ratios represents high-risk haplotype (C-C-T and/or C-C-C) relative to sum of common low-risk haplotypes A-T-C and C-T-C (reference). For EBV-Positive NPC cases, the reference group includes all non-NPC controls for each individual study (present cohort, Xu et al., Hui et al., Lam et al.). Odds ratios for select non-NPC hematologic malignancies use all other non-NPC controls as the reference group.
EBV, Epstein-Barr Virus; NPC, nasopharyngeal carcinoma; NHL, non-Hodgkin lymphoma, ALL, acute lymphoblastic leukemia; NKTCL, NK/T-cell lymphoma; AML, acute myeloid leukemia; MPN/MDS, myeloproliferative neoplasm/myelodysplastic syndrome.
*One patient with EBV-positive T-ALL had a single specimen positive for V700, V700L, and I613V (EBNA-1 viral load <100 IU/mL; V700, V700L, and I613V C_t 40.0, 40.3, 39.1), suggesting multiple infections. Sequencing of this specimen had low coverage, with a single V700L read, two I613 reads, and two V317 reads (haplotype [A/C]-C-C).

Supplementary Table S17. Association Between Observed EBV *BALF2* Single Nucleotide Variants and NPC in Genotyped Specimens.

Single Nucleotide Variant	Variant Frequency, NPC Cases	Variant Frequency, Non-NPC Controls	Odds Ratio and 95% CI (NPC vs. Non-NPC)	P-Value
162147G>A	20%	20%	1.00 (0.02-11.21)	1.00E+00
162195A>C	43%	7%	10.71 (2.30-47.14)	8.65E-04
162215C>A (V700L)	8%	11%	0.74 (0.08-3.48)	1.00E+00
162237C>G	50%	10%	8.73 (2.12-34.76)	9.21E-04
162273C>T	19%	46%	0.27 (0.05-1.06)	5.63E-02
162298A>G	0%	5%	0.00 (0.00-6.07)	1.00E+00
162464G>A	53%	10%	10.00 (2.88-34.21)	5.87E-05
162476T>C (I613V)	75%	7%	39.27 (11.57-141.19)	1.35E-12*
162507C>T	26%	35%	0.65 (0.17-2.10)	6.03E-01
162577T>C	0%	7%	0.00 (0.00-6.75)	1.00E+00
162675T>C	100%	96%	Undefined	1.00E+00
162681G>A	6%	3%	1.65 (0.03-17.93)	5.20E-01
162780A>C	0%	3%	0.00 (0.00-9.70)	1.00E+00
162852G>T	31%	42%	0.64 (0.16-2.19)	5.85E-01
162885G>A	19%	46%	0.27 (0.05-1.07)	5.58E-02
162963A>G	94%	95%	0.82 (0.08-41.55)	1.00E+00
163074C>T	6%	5%	1.38 (0.03-15.26)	5.78E-01
163107A>C	41%	11%	5.74 (1.47-21.16)	5.02E-03
163145G>A	0%	6%	0.00 (0.00-4.00)	5.94E-01
163206C>T	5%	5%	0.95 (0.02-8.53)	1.00E+00
163283C>T	0%	4%	0.00 (0.00-10.06)	1.00E+00
163287G>A	53%	7%	14.20 (3.63-54.99)	2.13E-05*
163293G>A	29%	28%	1.05 (0.27-3.54)	1.00E+00
163364C>T (V317M)	63%	1%	127.50 (22.68-1,182.58)	6.16E-14*
163377A>G	78%	48%	3.76 (1.08-16.50)	2.30E-02
163404C>A	50%	11%	7.75 (0.85-65.65)	3.62E-02
163422G>T	60%	10%	13.50 (1.24-175.59)	1.53E-02
163458T>C	80%	53%	3.57 (0.33-180.75)	3.69E-01
163464G>A	60%	1%	103.50 (4.56-5,208.38)	5.80E-04

Includes single nucleotide variants observed in at least 3 patients. NPC, nasopharyngeal carcinoma; CI, confidence interval.
*Exceeds Bonferroni-corrected statistical significance threshold when adjusting for multiple hypothesis testing.

Supplementary Table S18. Association Between Observed EBV *BALF2* Single Nucleotide Variants and *BALF2* Haplotypes

Single Nucleotide Variant	Variant Frequency H-H-L Haplotype	Variant Frequency H-H-H Haplotype	Variant Frequency Low-Risk Haplotypes	P-Value (H-H-L vs. Low-Risk Haplotypes)	P-Value (H-H-H vs. Low-Risk Haplotypes)
162147G>A	0%	0%	25%	1.89E-01	1.00E+00
162195A>C	36%	100%	2%	1.04E-03	2.60E-09*
162237C>G	82%	100%	2%	1.27E-09*	2.60E-09*
162273C>T	9%	0%	49%	1.14E-02	3.81E-03
162298A>G	18%	0%	4%	1.25E-01	1.00E+00
162464G>A	85%	100%	0%	2.81E-13*	4.24E-14*
162507C>T	15%	20%	40%	1.26E-01	3.12E-01
162577T>C	0%	0%	8%	1.00E+00	1.00E+00
162675T>C	100%	100%	96%	1.00E+00	1.00E+00
162681G>A	42%	0%	0%	8.61E-06*	1.00E+00
162780A>C	17%	0%	2%	6.46E-02	1.00E+00
162852G>T	42%	20%	44%	1.00E+00	1.86E-01
162885G>A	25%	0%	48%	2.16E-01	4.44E-03
162963A>G	100%	100%	95%	1.00E+00	1.00E+00
163074C>T	36%	11%	0%	1.29E-04	1.02E-01
163107A>C	46%	100%	3%	1.05E-04	1.48E-09*
163145G>A	15%	0%	5%	2.08E-01	1.00E+00
163206C>T	38%	9%	1%	7.46E-05	2.03E-01
163283C>T	31%	0%	0%	1.50E-04	1.00E+00
163287G>A	54%	100%	0%	7.54E-08*	4.69E-14*
163293G>A	23%	10%	34%	5.41E-01	1.65E-01
163377A>G	85%	100%	47%	1.61E-02	6.67E-04
163404C>A	67%	100%	0%	7.01E-07*	1.21E-07*
163422G>T	56%	100%	0%	1.21E-05*	1.31E-07*
163458T>C	78%	100%	49%	1.57E-01	5.52E-02
163464G>A	0%	80%	0%	1.00E+00	7.87E-06*

Includes single nucleotide variants observed in at least 3 patients, excluding variants that define haplotypes (162215C>A [V700L], 162476T>C [I613V], 163364C>T [V317M].
H-H-L haplotype is defined by V700, I613V, and V317. H-H-H haplotype is defined by V700, I613V, and V317M. All other haplotypes are considered low-risk for NPC.
NPC, nasopharyngeal carcinoma; CI, confidence interval.
*Exceeds Bonferroni-corrected statistical significance threshold when adjusting for multiple hypothesis testing.

Supplementary Table S19. Base Case Resource Utilization, NPC Mortality Reduction, and Cost-Effectiveness among Men and Women for Variant-Agnostic and *BALF2* Variant-Informed Screening Strategies. For a hypothetical cohort of 50-year-old men and women who develop NPC in southern China under base case assumptions, 10-year survival improved from 70.4% (95% CI 68.1-72.5%) in an unscreened cohort to a median of 85.7% (range, 85.4-87.0%) with variant-agnostic screening and 85.2% (range, 84.3-85.9%) with variant-informed screening). This corresponded to a median 10-year reduction in NPC-specific death of 51.8% (range, 50.7-56.3%) with variant-agnostic screening and 47.7% (range, 47.1-52.3%) with *BALF2* variant-informed screening. In the highest incidence region, the 7% relative reduction in screening sensitivity after *BALF2* triage resulted in approximately 3.4 excess NPC deaths per 100,000 after onetime screening. For strategies A_0-C_0 and G_0, the corresponding variant-informed strategies reduced screening costs by a median of 25.7% (range, 0.1-49.8%). For strategies D_0-F_0, the corresponding variant-informed strategies increased costs by a median of 17.2% (range, 5.1-35.8%). Although per-subject screening costs were slightly increased with strategies D_{BALF2}-F_{BALF2}, total incremental costs were lower after integrating all costs of screening, work-up, and treatment.

Variant-informed screening reduced referrals for endoscopy and/or MRI by approximately 40% relative to the corresponding variant-agnostic strategy, which is equal to the population prevalence of low-risk *BALF2* haplotypes. This reduction in referrals for the second and third steps of screening averted a median of 2,969 screening visits per 100,000 subjects (range, 35-5,459). At the ICER/GDP_{ppp}≤2.0 willingness-to-pay (WTP) threshold, screening was cost-effective in all populations except Hengdong, China (due to lower NPC incidence). Variant-informed screening typically offered similar ICERs due to slightly reduced screening sensitivity (7%), a slight increase in laboratory costs, and a 40% reduction in referrals for endoscopy/MRI.

Strategy ID	Screening Strategy	Resource Utilization Per 100,000 Screened Subjects*			Cost-Effectiveness**				
		Endoscopies	MRIs	Total Screening Visits	NPC Deaths Per 100,000	Incremental Cost ($I)	ICER/GDP_{ppp}	ICER/GDP_{ppp} ≤ 2.0	ICER/GDP_{ppp} ≤ 1.0
	No Screening	-	-	-	185.22 (87.39-452.15)	-	-	-	-
A_0	Plasma EBV BamHI-W DNA PCR→<br>Endoscopy	5507 (5495-5523)	0 (0-0)	104783 (104557-105097)	172.30 (78.56-419.79)	34.37 (34.15-65.41)	1.12 (0.35-2.38)	11/12 (92%)	5/12 (42%)
A_{BALF2}	Plasma EBV BamHI-W DNA PCR+BALF2 PCR→<br>Endoscopy	3311 (3304-3321)	0 (0-0)	102587 (102366-102895)	173.67 (79.50-423.23)	32.63 (32.40-57.11)	1.15 (0.35-2.60)	11/12 (92%)	5/12 (42%)
B_0	Plasma EBV BamHI-W DNA PCR→<br>MRI nasopharynx	2044 (2039-2050)	5472 (5460-5489)	106792 (106562-107112)	171.46 (77.98-417.67)	36.28 (35.45-72.11)	1.13 (0.36-2.33)	11/12 (92%)	5/12 (42%)
B_{BALF2}	Plasma EBV BamHI-W DNA PCR+BALF2 PCR→<br>MRI nasopharynx	1236 (1234-1240)	3311 (3304-3321)	103823 (103600-104135)	172.83 (78.92-421.12)	33.53 (32.72-59.89)	1.12 (0.34-2.47)	11/12 (92%)	5/12 (42%)
C_0	Plasma EBV BamHI-W DNA PCR→<br>Endoscopy+MRI nasopharynx	5472 (5460-5489)	5472 (5460-5489)	110220 (109983-110551)	171.46 (77.98-417.67)	40.02 (39.20-88.34)	1.29 (0.43-2.57)	11/12 (92%)	5/12 (42%)
C_{BALF2}	Plasma EBV BamHI-W DNA PCR+BALF2 PCR→<br>Endoscopy+MRI nasopharynx	3311 (3304-3321)	3311 (3304-3321)	105897 (105670-106215)	172.83 (78.92-421.12)	35.80 (34.99-68.69)	1.25 (0.39-2.63)	11/12 (92%)	5/12 (42%)
D_0	Plasma EBV BamHI-W DNA PCR→<br>Plasma EBV BamHI-W DNA PCR→<br>Endoscopy	1520 (1516-1524)	0 (0-0)	106305 (106076-106624)	172.30 (78.56-419.79)	31.06 (30.84-48.82)	0.89 (0.26-2.15)	11/12 (92%)	6/12 (50%)
D_{BALF2}	Plasma EBV BamHI-W DNA PCR+BALF2 PCR→<br>Plasma EBV BamHI-W DNA PCR→<br>Endoscopy	920 (918-923)	0 (0-0)	103507 (103284-103817)	173.67 (79.50-423.23)	30.44 (30.21-46.46)	0.98 (0.28-2.42)	11/12 (92%)	6/12 (50%)
E_0	Plasma EBV BamHI-W DNA PCR→<br>Plasma EBV BamHI-W DNA PCR→<br>MRI nasopharynx	568 (567-570)	1521 (1517-1525)	106839 (106610-107160)	171.46 (77.98-417.67)	31.15 (30.33-48.59)	0.84 (0.24-2.00)	11/12 (92%)	7/12 (58%)
E_{BALF2}	Plasma EBV BamHI-W DNA PCR+BALF2 PCR→<br>Plasma EBV BamHI-W DNA PCR→<br>MRI nasopharynx	344 (343-345)	920 (918-923)	103850 (103627-104162)	172.83 (78.92-421.12)	30.25 (29.44-46.11)	0.90 (0.25-2.22)	11/12 (92%)	6/12 (50%)
F_0	Plasma EBV BamHI-W DNA PCR→<br>Plasma EBV BamHI-W DNA PCR→<br>Endoscopy+MRI nasopharynx	1521 (1517-1525)	1521 (1517-1525)	107792 (107560-108116)	171.46 (77.98-417.67)	32.19 (31.37-52.49)	0.89 (0.26-2.07)	11/12 (92%)	7/12 (58%)
F_{BALF2}	Plasma EBV BamHI-W DNA PCR+BALF2 PCR→<br>Plasma EBV BamHI-W DNA PCR→<br>Endoscopy+MRI nasopharynx	920 (918-923)	920 (918-923)	104427 (104202-104740)	172.83 (78.92-421.12)	30.88 (30.07-47.89)	0.94 (0.27-2.27)	11/12 (92%)	6/12 (50%)
G_0	Serum EBV VCA IgA [>1:5]→<br>Nasopharyngeal EBV BamHI-W DNA PCR [mean+2SD]→<br>Endoscopy	90 (90-90)	0 (0-0)	104154 (103930-104467)	172.69 (78.83-420.76)	14.97 (14.72-26.14)	0.44 (0.14-1.06)	12/12 (100%)	11/12 (92%)
G_{BALF2}	Serum EBV VCA IgA [>1:5]→<br>Nasopharyngeal EBV BamHI-W DNA PCR<br>[mean+2SD]+BALF2 PCR→<br>Endoscopy	54 (54-55)	0 (0-0)	104118 (103894-104431)	173.83 (79.61-423.64)	14.93 (14.68-26.01)	0.49 (0.16-1.20)	12/12 (100%)	11/12 (92%)

Values are reported as median (range) or counts (percent) across the 12 high-risk populations in southern China, Hong Kong SAR, Macao SAR, Singapore, and the Republic of China.

NPC, nasopharyngeal carcinoma; PCR, polymerase chain reaction; ELISA, enzyme-linked immunosorbent assay; MRI, magnetic resonance imaging; $I, 2021 international dollars; ICER/GDP_{ppp}, incremental cost-effectiveness ratio divided by the purchasing power parity-adjusted gross domestic product per capita in the population of interest. Willingness to pay thresholds of 2.0 and 1.0 were evaluated.

*Median (range) resource utilization per 100,000 screened subjects. For example, 5.5% of individuals screen positive by plasma *BamHI-W* PCR and undergo immediate nasoendoscopy for strategy A_0, yielding 5,507 endoscopies per 100,000 screened subjects. Total screening visits refers to all unique in-person screening visits. This is equal to the sum of plasma PCR, NP PCR, ELISA, MRI, and exam+endoscopy. *BALF2* qPCR is performed from residual extracted nucleic acids, and does not require an additional visit for specimen collection.

**Measures of cost-effectiveness for each onetime screening strategy across the 12 included populations, including median (range) incremental cost per screened subject, median (range) NPC deaths over lifetime horizon, median (range) ICER/GDP_{ppp}, and the number (percent) of screened populations which were cost-effective for each strategy at two willingness to pay thresholds (1.0 and 2.0 ICER/GDP_{ppp}).

Supplementary Table S20. Base Case Resource Utilization, NPC Mortality Reduction, and Cost-Effectiveness among Only Men for Variant-Agnostic and *BALF2* Variant-Informed Screening Strategies. Screening was more cost-effective when limited only to men due to higher incidence.

Strategy ID	Screening Strategy	Resource Utilization Per 100,000 Screened Subjects*			Cost-Effectiveness**				
		Endoscopies	MRIs	Total Screening Visits	NPC Deaths Per 100,000	Incremental Cost ($I)	$ICER/GDP_{ppp}$	$ICER/GDP_{ppp} \leq 2.0$	$ICER/GDP_{ppp} \leq 1.0$
	No Screening	-	-	-	274.90 (118.56-674.12)	-	-	-	-
A_0	Plasma EBV BamHI-W DNA PCR→ Endoscopy	5490 (5473-5517)	0 (0-0)	104475 (104149-104985)	256.06 (105.64-625.02)	34.56 (34.14-65.92)	0.85 (0.23-1.74)	12/12 (100%)	10/12 (83%)
A_{BALF2}	Plasma EBV BamHI-W DNA PCR+BALF2 PCR→ Endoscopy	3301 (3291-3317)	0 (0-0)	102286 (101966-102785)	258.06 (107.02-630.27)	32.82 (32.40-57.65)	0.89 (0.23-1.91)	12/12 (100%)	8/12 (67%)
B_0	Plasma EBV BamHI-W DNA PCR→ MRI nasopharynx	2038 (2031-2047)	5456 (5439-5483)	106479 (106146-106998)	241.98 (104.78-621.76)	36.20 (34.85-71.16)	0.86 (0.22-1.69)	12/12 (100%)	9/12 (75%)
B_{BALF2}	Plasma EBV BamHI-W DNA PCR+BALF2 PCR→ MRI nasopharynx	1233 (1229-1239)	3301 (3291-3317)	103519 (103195-104024)	256.83 (106.18-627.06)	33.46 (32.13-59.05)	0.85 (0.21-1.80)	12/12 (100%)	10/12 (83%)
C_0	Plasma EBV BamHI-W DNA PCR→ Endoscopy+MRI nasopharynx	5456 (5439-5483)	5456 (5439-5483)	109897 (109554-110434)	241.98 (104.78-621.76)	39.93 (38.58-87.38)	0.95 (0.27-1.87)	12/12 (100%)	7/12 (58%)
C_{BALF2}	Plasma EBV BamHI-W DNA PCR+BALF2 PCR→ Endoscopy+MRI nasopharynx	3301 (3291-3317)	3301 (3291-3317)	105587 (105257-106102)	256.83 (106.18-627.06)	35.72 (34.38-67.84)	0.94 (0.25-1.92)	12/12 (100%)	8/12 (67%)
D_0	Plasma EBV BamHI-W DNA PCR→ Plasma EBV BamHI-W DNA PCR→ Endoscopy	1515 (1511-1523)	0 (0-0)	105993 (105662-106511)	256.06 (105.64-625.02)	31.26 (30.84-49.37)	0.68 (0.17-1.57)	12/12 (100%)	10/12 (83%)
D_{BALF2}	Plasma EBV BamHI-W DNA PCR+BALF2 PCR→ Plasma EBV BamHI-W DNA PCR→ Endoscopy	917 (914-922)	0 (0-0)	103203 (102880-103707)	258.06 (107.02-630.27)	30.64 (30.21-46.92)	0.74 (0.19-1.78)	12/12 (100%)	10/12 (83%)
E_0	Plasma EBV BamHI-W DNA PCR→ Plasma EBV BamHI-W DNA PCR→ MRI nasopharynx	566 (564-569)	1516 (1511-1524)	106526 (106193-107046)	241.98 (104.78-621.76)	31.08 (29.74-47.91)	0.63 (0.15-1.46)	12/12 (100%)	10/12 (83%)
E_{BALF2}	Plasma EBV BamHI-W DNA PCR+BALF2 PCR→ Plasma EBV BamHI-W DNA PCR→ MRI nasopharynx	343 (341-344)	917 (914-922)	103546 (103222-104051)	256.83 (106.18-627.06)	30.18 (28.85-45.44)	0.68 (0.16-1.62)	12/12 (100%)	10/12 (83%)
F_0	Plasma EBV BamHI-W DNA PCR→ Plasma EBV BamHI-W DNA PCR→ Endoscopy+MRI nasopharynx	1516 (1511-1524)	1516 (1511-1524)	107476 (107140-108001)	241.98 (104.78-621.76)	32.12 (30.78-51.64)	0.67 (0.16-1.50)	12/12 (100%)	10/12 (83%)
F_{BALF2}	Plasma EBV BamHI-W DNA PCR+BALF2 PCR→ Plasma EBV BamHI-W DNA PCR→ Endoscopy+MRI nasopharynx	917 (914-922)	917 (914-922)	104120 (103795-104629)	256.83 (106.18-627.06)	30.81 (29.48-47.22)	0.71 (0.17-1.66)	12/12 (100%)	10/12 (83%)
G_0	Serum EBV VCA IgA [>1:5]→ Nasopharyngeal EBV BamHI-W DNA PCR [mean+2SD]→ Endoscopy	90 (89-90)	0 (0-0)	103849 (103524-104355)	256.62 (106.03-626.51)	15.22 (14.76-26.36)	0.34 (0.09-0.78)	12/12 (100%)	12/12 (100%)
G_{BALF2}	Serum EBV VCA IgA [>1:5]→ Nasopharyngeal EBV BamHI-W DNA PCR [mean+2SD]+BALF2 PCR→ Endoscopy	54 (54-55)	0 (0-0)	103813 (103489-104320)	258.30 (107.20-630.93)	15.18 (14.72-26.24)	0.38 (0.10-0.88)	12/12 (100%)	12/12 (100%)

Values are reported as median (range) or counts (percent) across the 12 high-risk populations in southern China, Hong Kong SAR, Macao SAR, Singapore, and the Republic of China.

NPC, nasopharyngeal carcinoma; PCR, polymerase chain reaction; ELISA, enzyme-linked immunosorbent assay; MRI, magnetic resonance imaging; $I, 2021 international dollars; $ICER/GDP_{ppp}$, incremental cost-effectiveness ratio divided by the purchasing power parity-adjusted gross domestic product per capita in the population of interest. Willingness to pay thresholds of 2.0 and 1.0 were evaluated.

*Median (range) resource utilization per 100,000 screened subjects. For example, 5.5% of individuals screen positive by plasma *BamHI-W* PCR and undergo immediate nasoendoscopy for strategy A_0, yielding 5,507 endoscopies per 100,000 screened subjects. Total screening visits refers to all unique in-person screening visits. This is equal to the sum of plasma PCR, NP PCR, ELISA, MRI, and exam+endoscopy. *BALF2* qPCR is performed from residual extracted nucleic acids, and does not require an additional visit for specimen collection.

**Measures of cost-effectiveness for each onetime screening strategy across the 12 included populations, including median (range) incremental cost per screened subject, median (range) NPC deaths over lifetime horizon, median (range) $ICER/GDP_{ppp}$, and the number (percent) of screened populations which were cost-effective for each strategy at two willingness to pay thresholds (1.0 and 2.0 $ICER/GDP_{ppp}$).

Supplementary Table S21. Cost-Effectiveness for Variant-Agnostic and *BALF2* Variant-Informed Screening Strategies with Variable Screening Age and Interval. Initial screening age was varied from 40-60 in five-year increments, and screening interval was varied from every 1-5 years in addition to once-lifetime screening. All screening ended after age 70. Across the 12 populations and 18 screening strategies, an initial screening age of 40 or 45 tended to be most cost-effective irrespective of screening interval. Interval screening was never as cost-effective as once-lifetime screening, commensurate with the prevalence of undiagnosed preclinical NPC. However, screening intervals as short as every two years could be cost-effective in the majority of populations and screening strategies. As screening frequency increased, the absolute number of NPC deaths averted increased while per-screen mortality reduction decreased. Variant-informed and variant-agnostic screening had similar ICERs with once-lifetime screening, whereas variant-informed screening became more cost-effective as the number of lifetime screens increased. This was due to the increasing proportion of individuals known to have low-risk *BALF2* haplotypes that were never subsequently screened.

Screening Frequency	Median (Range) ICER/GDP_{ppp}					Proportion of Scenarios Cost-Effective*				
	Initial Screening Age					Initial Screening Age				
**Men and Women**	**40**	**45**	**50**	**55**	**60**	**40**	**45**	**50**	**55**	**60**
Variant-Agnostic Screening										
Once Lifetime	0.84 (0.11-2.83)	0.72 (0.12-3.27)	0.97 (0.14-2.88)	1.08 (0.21-3.81)	1.77 (0.22-11.48)	92.6%	92.6%	90.7%	81.5%	58.3%
Every Five Years	1.41 (0.21-4.58)	1.45 (0.22-4.42)	1.59 (0.24-5.16)	1.80 (0.27-6.00)	2.28 (0.27-10.01)	66.7%	67.6%	61.1%	55.6%	41.7%
Every Four Years	1.60 (0.24-5.16)	1.73 (0.26-5.18)	1.80 (0.26-5.81)	2.18 (0.31-7.23)	3.01 (0.34-11.96)	63.0%	58.3%	55.6%	48.1%	33.3%
Every Three Years	1.62 (0.24-4.82)	1.80 (0.27-5.36)	1.98 (0.28-6.36)	2.27 (0.31-7.68)	3.35 (0.39-12.59)	61.1%	55.6%	50.9%	44.4%	29.6%
Every Two Years	1.81 (0.27-5.52)	1.97 (0.29-5.85)	2.14 (0.31-6.85)	2.68 (0.35-8.85)	3.39 (0.37-12.35)	53.7%	51.9%	46.3%	35.2%	28.7%
Every One Year	2.41 (0.36-7.34)	2.58 (0.38-7.78)	2.85 (0.41-9.27)	3.46 (0.43-12.00)	4.42 (0.48-17.74)	39.8%	38.0%	32.4%	25.0%	15.7%
BALF2 Variant-Informed Screening										
Once Lifetime	0.87 (0.12-2.78)	0.72 (0.13-3.22)	1.00 (0.16-2.85)	1.09 (0.23-3.81)	1.84 (0.25-11.61)	92.6%	92.6%	92.6%	80.6%	53.7%
Every Five Years	1.45 (0.23-4.23)	1.36 (0.24-4.13)	1.55 (0.27-4.92)	1.80 (0.30-5.84)	2.51 (0.30-10.01)	67.6%	67.6%	57.4%	54.6%	38.9%
Every Four Years	1.58 (0.26-4.68)	1.60 (0.28-4.75)	1.69 (0.29-5.47)	2.13 (0.35-6.95)	3.19 (0.39-11.90)	63.0%	57.4%	55.6%	48.1%	30.6%
Every Three Years	1.57 (0.26-4.28)	1.62 (0.30-4.84)	1.84 (0.31-5.85)	2.21 (0.34-7.28)	3.52 (0.43-12.43)	63.9%	56.5%	51.9%	45.4%	27.8%
Every Two Years	1.66 (0.29-4.76)	1.74 (0.32-5.13)	1.92 (0.34-6.15)	2.52 (0.38-8.16)	3.54 (0.41-12.03)	59.3%	55.6%	51.9%	38.9%	27.8%
Every One Year	2.02 (0.38-5.93)	2.11 (0.41-6.43)	2.35 (0.45-7.88)	3.06 (0.47-10.67)	4.38 (0.53-16.66)	49.1%	47.2%	38.0%	26.9%	18.5%
**Men Only**	**40**	**45**	**50**	**55**	**60**	**40**	**45**	**50**	**55**	**60**
Variant-Agnostic Screening										
Once Lifetime	0.56 (0.08-1.86)	0.50 (0.08-2.40)	0.73 (0.09-2.10)	0.78 (0.15-3.19)	1.29 (0.23-10.12)	100.0%	97.2%	99.1%	92.6%	76.9%
Every Five Years	1.06 (0.15-3.07)	1.02 (0.16-3.28)	1.14 (0.18-4.01)	1.34 (0.23-5.04)	1.70 (0.28-10.14)	82.4%	81.5%	82.4%	75.9%	62.0%
Every Four Years	1.17 (0.17-3.49)	1.21 (0.19-3.99)	1.25 (0.20-4.51)	1.55 (0.26-5.96)	2.16 (0.32-14.00)	79.6%	76.9%	75.9%	65.7%	46.3%
Every Three Years	1.18 (0.17-3.52)	1.24 (0.20-4.10)	1.38 (0.23-4.98)	1.63 (0.26-6.49)	2.42 (0.33-16.36)	79.6%	76.9%	71.3%	63.9%	41.7%
Every Two Years	1.32 (0.19-3.87)	1.36 (0.22-4.62)	1.45 (0.25-5.33)	1.88 (0.29-7.71)	2.43 (0.32-13.91)	75.0%	69.4%	65.7%	52.8%	42.6%
Every One Year	1.75 (0.26-5.22)	1.77 (0.28-6.00)	1.94 (0.34-7.26)	2.39 (0.37-11.15)	3.19 (0.40-16.55)	57.4%	55.6%	50.9%	44.4%	33.3%
BALF2 Variant-Informed Screening										
Once Lifetime	0.58 (0.09-1.83)	0.51 (0.09-2.37)	0.73 (0.10-2.08)	0.77 (0.17-3.20)	1.42 (0.26-10.33)	100.0%	94.4%	99.1%	92.6%	72.2%
Every Five Years	0.97 (0.17-2.84)	0.93 (0.18-3.08)	1.07 (0.21-3.84)	1.26 (0.26-4.93)	1.80 (0.32-10.29)	83.3%	83.3%	79.6%	70.4%	54.6%
Every Four Years	1.04 (0.19-3.18)	1.08 (0.21-3.69)	1.16 (0.23-4.26)	1.49 (0.29-5.78)	2.31 (0.36-14.34)	80.6%	77.8%	76.9%	60.2%	44.4%
Every Three Years	1.05 (0.19-3.15)	1.09 (0.23-3.73)	1.26 (0.25-4.63)	1.56 (0.29-6.24)	2.53 (0.37-16.79)	81.5%	76.9%	71.3%	60.2%	41.7%
Every Two Years	1.09 (0.21-3.35)	1.17 (0.24-4.08)	1.31 (0.28-4.82)	1.77 (0.32-7.27)	2.51 (0.36-13.72)	78.7%	75.9%	67.6%	53.7%	44.4%
Every One Year	1.34 (0.28-4.23)	1.41 (0.30-4.99)	1.59 (0.37-6.23)	2.07 (0.40-10.09)	3.10 (0.44-15.58)	68.5%	64.8%	54.6%	48.1%	36.1%

Values are reported as median (range) or percentages across the 18 screening strategies (9 variant-agnostic, 9 variant-informed) and 12 high-risk populations in southern China, Hong Kong SAR, Macao SAR, Singapore, and the Republic of China.

ICER/GDP_{ppp}, incremental cost-effectiveness ratio divided by the purchasing power parity-adjusted gross domestic product per capita in the population of interest.

*Cost-effective defined as willingness to pay threshold of 2.0 ICER/GDP_{ppp}.

Supplementary Table S22. Base Case Cost-Effectiveness and Probabilistic Sensitivity Analysis for Example Population of 50-Year Old Men and Women Screened Once in Guangzhou, China. Probabilistic sensitivity analysis (PSA) assessed uncertainty around base case estimates. All base case estimates for NPC mortality reduction, incremental costs, and ICERs were within the PSA 95% confidence intervals. Median ICERs from PSA were slightly lower than base case estimates. In Guangzhou, nearly 100% of simulations were below the ICER/GDP$_{ppp}$ ≤ 2.0 WTP threshold, whereas 38-100% of simulations were below a WTP threshold of 1.0, depending on screening strategy.

Strategy ID	Screening Strategy	Base Case			Probabilistic Sensitivity Analysis					
		NPC Deaths Averted per 100,000	Incremental Cost	ICER/GDP$_{ppp}$	NPC Deaths Averted per 100,000	Incremental Cost	ICER/GDP$_{ppp}$	Simulations ICER/GDP$_{ppp}$ ≤ 2.0	Simulations ICER/GDP$_{ppp}$ ≤ 1.0	Simulations ICER/GDP$_{ppp}$ ≤ 0.5
A_0	Plasma EBV BamHI-W DNA PCR→ Endoscopy	16.40	34.22	1.28	20.65 (14.66-26.17)	33.05 (21.64-46.27)	0.90 (0.56-1.66)	100%	63%	2%
A_{BALF2}	Plasma EBV BamHI-W DNA PCR+BALF2 PCR→ Endoscopy	14.66	32.47	1.40	18.39 (13.31-23.55)	32.26 (21.78-36.51)	0.97 (0.64-1.71)	99%	52%	0%
B_0	Plasma EBV BamHI-W DNA PCR→ MRI nasopharynx	17.48	36.08	1.24	21.90 (15.44-27.63)	33.84 (23.85-48.43)	0.88 (0.52-1.59)	100%	64%	4%
B_{BALF2}	Plasma EBV BamHI-W DNA PCR+BALF2 PCR→ MRI nasopharynx	15.73	33.33	1.32	19.79 (14.08-25.16)	32.95 (23.43-37.53)	0.93 (0.60-1.64)	100%	59%	0%
C_0	Plasma EBV BamHI-W DNA PCR→ Endoscopy+MRI nasopharynx	17.48	39.82	1.37	21.90 (15.44-27.63)	37.87 (25.61-52.46)	0.97 (0.58-1.76)	100%	56%	1%
C_{BALF2}	Plasma EBV BamHI-W DNA PCR+BALF2 PCR→ Endoscopy+MRI nasopharynx	15.73	35.60	1.41	19.79 (14.08-25.16)	35.06 (25.28-39.73)	1.00 (0.65-1.73)	99%	49%	0%
D_0	Plasma EBV BamHI-W DNA PCR→ Plasma EBV BamHI-W DNA PCR→ Endoscopy	16.40	30.91	1.16	20.65 (14.66-26.17)	27.02 (6.70-57.22)	0.76 (0.15-2.04)	96%	68%	27.
D_{BALF2}	Plasma EBV BamHI-W DNA PCR+BALF2 PCR→ Plasma EBV BamHI-W DNA PCR→ Endoscopy	14.66	30.28	1.31	18.39 (13.31-23.55)	30.01 (19.89-35.02)	0.89 (0.61-1.63)	100%	58%	0%
E_0	Plasma EBV BamHI-W DNA PCR→ Plasma EBV BamHI-W DNA PCR→ MRI nasopharynx	17.48	30.95	1.07	21.90 (15.44-27.63)	27.19 (6.39-58.03)	0.72 (0.13-1.88)	98%	71%	30%
E_{BALF2}	Plasma EBV BamHI-W DNA PCR+BALF2 PCR→ Plasma EBV BamHI-W DNA PCR→ MRI nasopharynx	15.73	30.05	1.19	19.79 (14.08-25.16)	29.91 (19.63-34.72)	0.82 (0.53-1.50)	100%	73%	3%
F_0	Plasma EBV BamHI-W DNA PCR→ Plasma EBV BamHI-W DNA PCR→ Endoscopy+MRI nasopharynx	17.48	31.99	1.10	21.90 (15.44-27.63)	27.85 (6.70-59.51)	0.74 (0.13-1.95)	98%	70%	29%
F_{BALF2}	Plasma EBV BamHI-W DNA PCR+BALF2 PCR→ Plasma EBV BamHI-W DNA PCR→ Endoscopy+MRI nasopharynx	15.73	30.68	1.21	19.79 (14.08-25.16)	30.37 (20.36-35.23)	0.84 (0.54-1.53)	100%	71%	2%
G_0	Serum EBV VCA IgA [>1:5]→ Nasopharyngeal EBV BamHI-W DNA PCR [mean+2SD]→ Endoscopy	15.91	14.80	0.58	19.96 (14.25-25.44)	14.66 (9.70-18.80)	0.40 (0.25-0.78)	100%	100%	64%
G_{BALF2}	Serum EBV VCA IgA [>1:5]→ Nasopharyngeal EBV BamHI-W DNA PCR [mean+2SD]+BALF2 PCR→ Endoscopy	14.45	14.76	0.65	18.08 (13.13-23.18)	14.62 (9.67-18.73)	0.44 (0.29-0.85)	100%	100%	59%

Base case results are reported for population of 50 year-old men and women screened once in Guangzhou, China. Corresponding results from probabilistic sensitivity analysis are reported as median (95% confidence interval) and percentages.
NPC, nasopharyngeal carcinoma; PCR, polymerase chain reaction; ELISA, enzyme-linked immunosorbent assay; MRI, magnetic resonance imaging; ICER/GDP$_{ppp}$, incremental cost-effectiveness ratio divided by the purchasing power parity-adjusted gross domestic product per capita in the population of interest.
Willingness to pay thresholds of 2.0, 1.0, and 0.5 were evaluated.

Supplementary Table S23. Population-Specific Results from Base Case and Sensitivity Analyses, Including Analyses by Gender, Initial Screening Age, and Screening Interval. Among the four incidence databases, 12 unique populations met inclusion criteria. The age-standardized rate per 100,000 person-years ranged from 3.6 in the Republic of China to 16.6 in Zhuhai, Guangdong. Per-capita PPP-adjusted GDP (GDP_{ppp}) ranged from $18,931 in China to $102,742 in Singapore.

See file, "Supplementary Table 23 - Population-Level Data.xlsx"

Supplementary Figure S1: Multiplex EBV *BALF2* genotyping qPCR design and example amplification curves. A) Regions within EBV BALF2 reference sequence (GenBank NC_00706.1:162397-163444) amplified by *BALF2* qPCR. Three sets of conserved primers flank the three variants (V700L, I613V, and V317M) which define low-risk and high-risk *BALF2* haplotypes. Four allele-specific hydrolysis probes targeting V700, V700L, I613V, and V317M and cleaved during primer extension. **B)** Example multiplex *BALF2* qPCR amplification plots demonstrating detection or absence of V700, V700L, I613V, and V317M from EBV-negative plasma, dsDNA low-risk and high-risk controls, wild-type EBV DNA from B95-8 cell line, and clinical specimens with A-T-C, C-C-C, and C-C-T *BALF2* haplotypes. Abbreviations: FWD, forward; REV, reverse; EBV, Epstein-Barr Virus; LLOD, lower limit of detection; dsDNA, double-stranded DNA; C_t, cycle threshold.

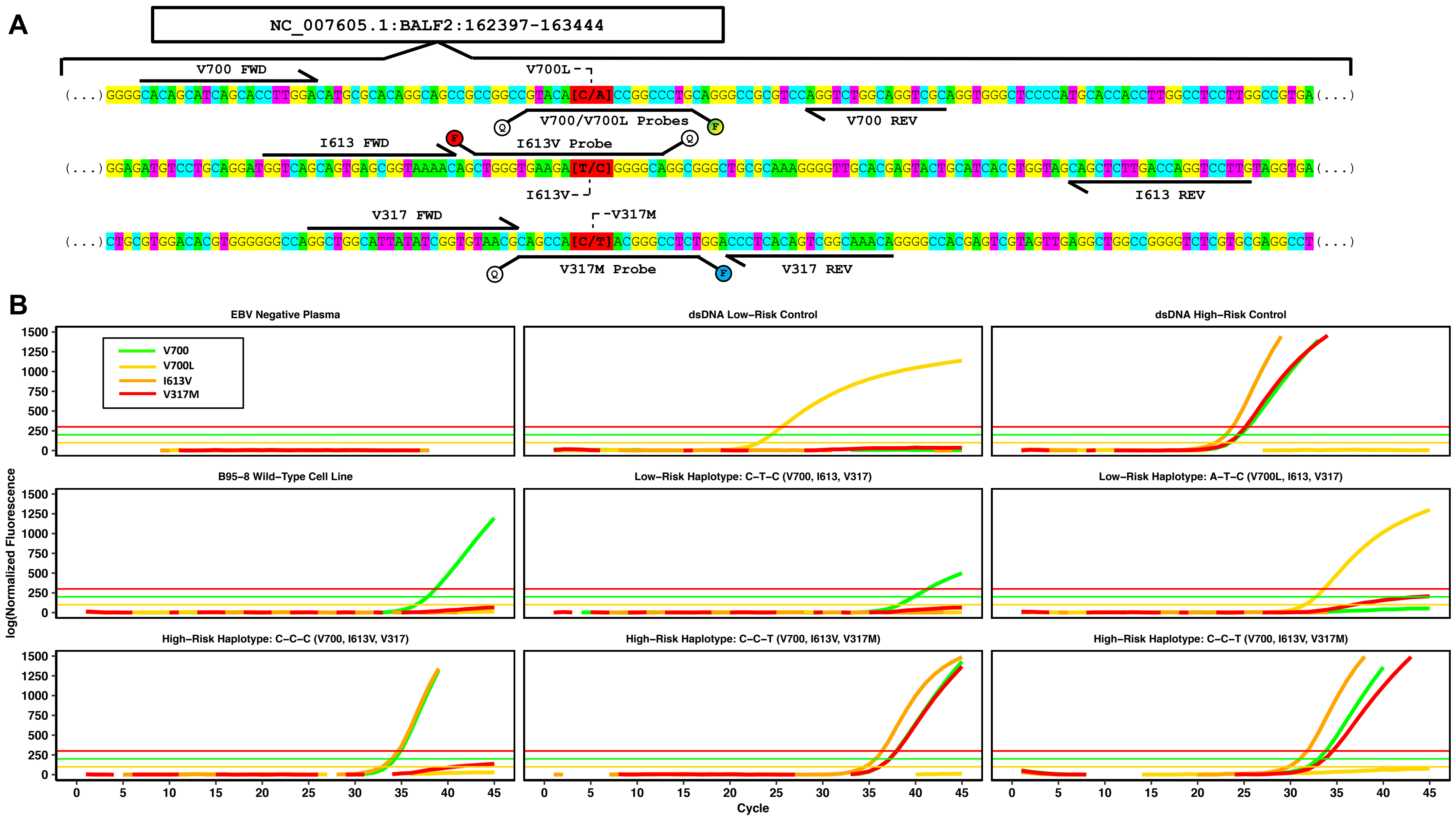

Supplementary Figure S2: Schematic of natural history Markov model of endemic nasopharyngeal carcinoma (NPC). Healthy adults could develop Epstein-Barr Virus-associated (EBV) stage I NPC and progress without detection to more advanced stages of undetected disease. This is the cohort of patients with prevalent undiagnosed NPC that can be screen-detected. Each undetected stage of NPC could present symptomatically or be detected by screening. A subset of healthy adults and those with each stage of preclinical NPC are biomarker positive or negative (for each of ten biomarker combinations). At the time of screening, participants who are biomarker positive (whether healthy or those with preclinical NPC) test positive and are found to be true positives or false positives. Similarly, participants who are biomarker negative are true negatives or false negatives. Each detected (incident) case of NPC is managed with radiotherapy and/or chemotherapy, and can either be cured or develop locoregional recurrence (LRR) or distant metastasis (DM) after treatment and die from their disease. Patients with stage IVC NPC receive chemotherapy until dying from their disease. In each state, there is also the risk of remaining within the same state or dying from other causes.

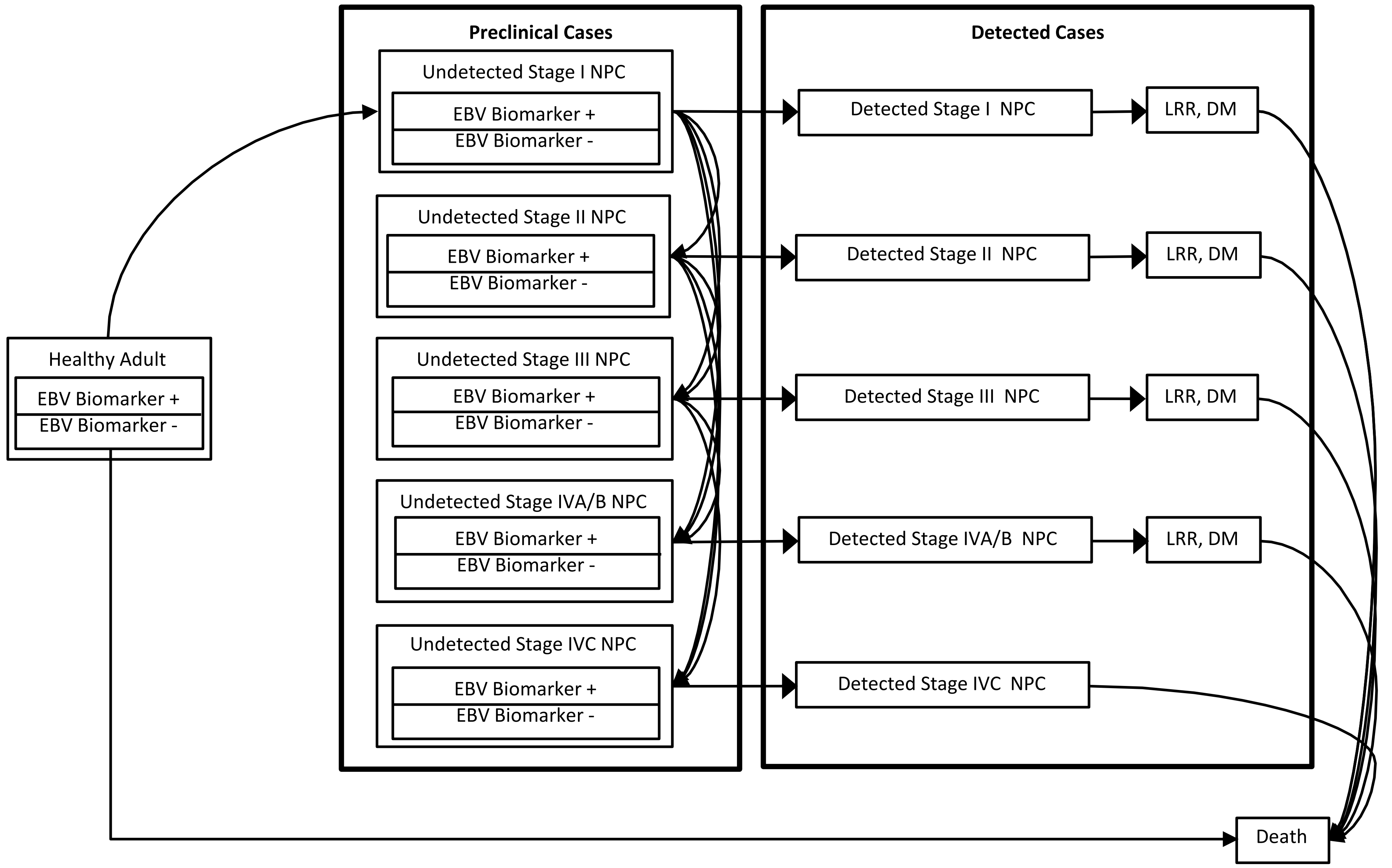

Supplementary Figure S3: Sensitivity analysis of modeled variant-informed screening strategies in endemic populations. A) The modeled number of NPC deaths averted per screening test per 100,000 screened individuals in a modeled population of men and women in southern China, screened with variant-agnostic or variant-informed screening strategies starting from ages 40-60 with screening frequency ranging from every 1-5 years. While absolute mortality reduction is greatest with more frequent screening, the relative mortality reduction per test is lower with more frequent screening due to the lower prevalence of undiagnostic NPC with each successive screen. Per-test mortality reduction is similar irrespective of initial screening age. **B)** Prevalence of known and unknown *BALF2* haplotypes in endemic population as the number of plasma PCR screens increase. Patients with low-risk haplotypes discontinue future screening. **C)** Results of deterministic sensitivity analysis indicating proportional change in cost-effectiveness (ICER/GDP_{ppp}) as model parameters vary within ranges of uncertainty. Top 18 parameters that impact cost-effectiveness are plotted, with upper/lower limits denoted by black/gray bars. Deterministic sensitivity analysis identified parameters that most impacted cost-effectiveness (Figure 4, Supplementary Tables 12 and 20). Within the studied parameter ranges, variations in stage-specific recurrence/survival rates, most health utilities, and the costs of imaging, workup, radiotherapy, and chemotherapy modestly impacted ICERs (±1.0-7.0%). Screening was more cost-effective (16.4% ICER decrease) in the setting of 2D/3DCRT owing to decreased survival, higher recurrence rates, and late toxicities that most impact patients with advanced-stage disease. Screening performance and costs were the principal determinants of cost-effectiveness. A 5.0% absolute decrease in sensitivity or compliance increased median ICER by 9.5% and 12.3%, respectively. A 0.05 increase or decrease in the long-term utility after definitive radiotherapy alone for stage I NPC decreased or increased median ICER by 16.0-23.6% Discount rate (0-5%) had the largest impact upon median ICER (±55%). Due to uncertainty in population-specific costs, we studied a broad range (50-200%) of screening costs. Doubling reagent/consumables costs or laboratory technician costs increased median ICER by 66.3% and 10.4%, respectively. We also studied uncertainty in the WHO-CHOICE regression estimates of healthcare costs in each economy, which had a <10% impact on median ICER. Because *BALF2* variant-informed screening strategies were typically cost-neutral compared with variant-agnostic screening, varying the prevalence of high-risk haplotypes above or below the 60.5% obtained from meta-analysis only impacted median ICER by 2.5%. Abbreviations: ICER, incremental cost-effectiveness ratio; GDP_{ppp}, purchasing power parity-adjusted per-capita gross domestic product; MRI, magnetic resonance imaging; PCR, polymerase chain reaction; ELISA, enzyme-linked immunosorbent assay; NED, no evidence of disease; IMRT, intensity-modulated radiotherapy; 2D/3D CRT, 2D/3D conformal radiotherapy; WHO, World Health Organization; OS, overall survival.

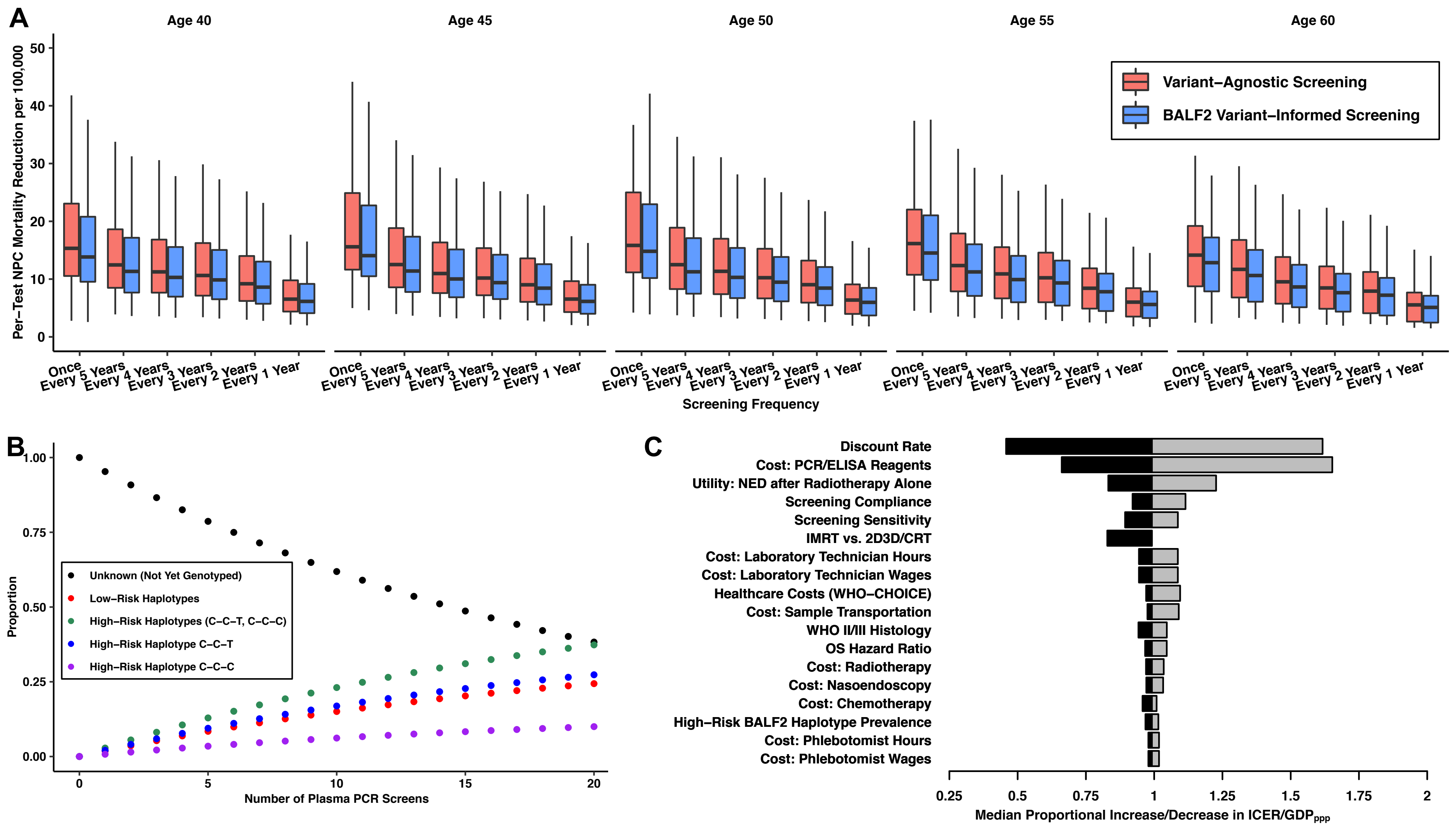

Supplement: Supplementary file 1 — Additional file 1. [file 12943_2022_1625_MOESM1_ESM.zip › 06.19.22_ebv_balf2_supplement final.pdf]
